# Nitrogen-doped graphene quantum dots induce ferroptosis through disrupting calcium homeostasis in microglia

**DOI:** 10.1186/s12989-022-00464-z

**Published:** 2022-03-24

**Authors:** Tianshu Wu, Xinyu Wang, Jin Cheng, Xue Liang, Yimeng Li, Min Chen, Lu Kong, Meng Tang

**Affiliations:** 1 Key Laboratory of Environmental Medicine and Engineering, Ministry of Education, School of Public Health, Southeast University, Nanjing, 210009 People’s Republic of China; 2 School of Public Health, Shandong First Medical University & Shandong Academy of Medical Sciences, Jinan, People’s Republic of China

**Keywords:** Hippocampus, Necroinflammation, L-VGCCs, RyR calcium channels, ER stress

## Abstract

**Background:**

Along with the wild applications of nitrogen-doped graphene quantum dots (N-GQDs) in the fields of biomedicine and neuroscience, their increasing exposure to the public and potential biosafety problem has gained more and more attention. Unfortunately, the understanding of adverse effects of N-GQDs in the central nervous system (CNS), considered as an important target of nanomaterials, is still limited.

**Results:**

After we found that N-GQDs caused cell death, neuroinflammation and microglial activation in the hippocampus of mice through the ferroptosis pathway, microglia was used to assess the molecular mechanisms of N-GQDs inducing ferroptosis because it could be the primary target damaged by N-GQDs in the CNS. The microarray data suggested the participation of calcium signaling pathway in the ferroptosis induced by N-GQDs. In microglial BV2 cells, when the calcium content above the homeostatic level caused by N-GQDs was reversed, the number of cell death, ferroptosis alternations and excessive inflammatory cytokines release were all alleviated. Two calcium channels of L-type voltage-gated calcium channels (L-VGCCs) in plasma membrane and ryanodine receptor (RyR) in endoplasmic reticulum (ER) took part in N-GQDs inducing cytosolic calcium overload. L-VGCCs and RyR calcium channels were also involved in promoting the excess iron influx and triggering ER stress response, respectively, which both exert excessive ROS generation and result in the ferroptosis and inflammation in BV2 cells.

**Conclusion:**

N-GQDs exposure caused ferroptosis and inflammatory responses in hippocampus of mice and cultured microglia through activating two calcium channels to disrupt intracellular calcium homeostasis. The findings not only posted an alert for biomedical applications of N-GQDs, but also highlighted an insight into mechanism researches of GQDs inducing multiple types of cell death in brain tumor therapy in the future.

**Graphical abstract:**

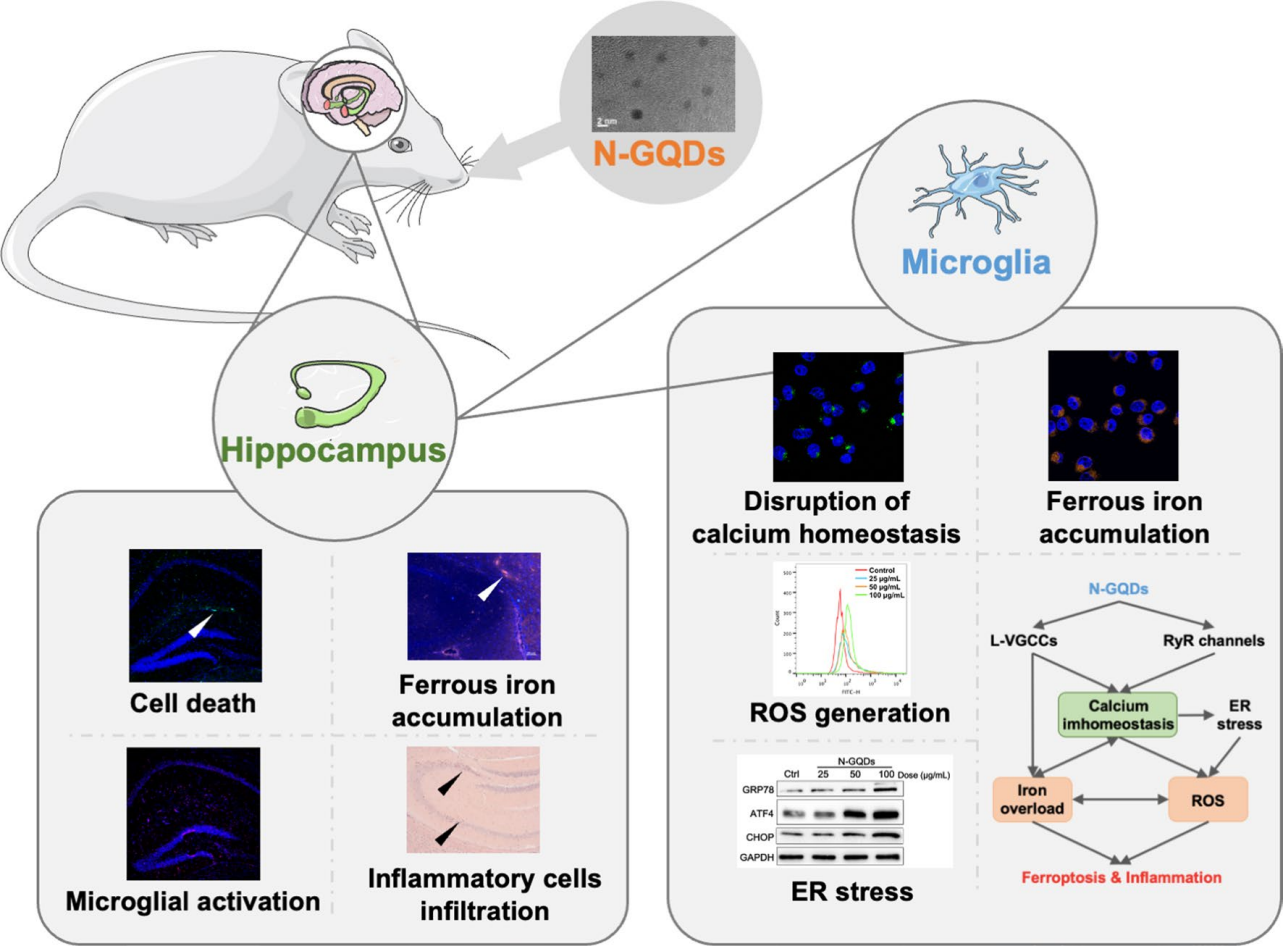

**Supplementary Information:**

The online version contains supplementary material available at 10.1186/s12989-022-00464-z.

## Background

A novel type of zero-dimensional carbon-based nanomaterials called graphene quantum dots (GQDs) has been considered as a promising nanomaterial for wide applications. Among multiple functionalizations of GQDs, the production of nitrogen-doped graphene quantum dots (N-GQDs) is an economical and green strategy in the use and development of a variety of applications due to their outstanding properties, including superior biocompatibility, bright fluorescence and strong resistance to photobleaching [1–3]. With the increasing applications of N-GQDs, their exposure to the public in the environment will pose a threat to human health. Recent studies have suggested that inhaled exposed carbon-based nanoparticles could reach the central nervous system (CNS) through the olfactory nerve or trigeminal nerve, and result in neurotoxic effects [4–6], which emphasizes the importance of assessing the neurotoxicity of N-GQDs. Additionally, GQDs-based neuroimaging techniques, photophysical therapies and drug delivery systems to the CNS have been considered by more and more researchers in present days [7–10]. Little information of the adverse impacts of N-GQDs on the CNS will impose a restriction on their contributions in the development of nanotheranostics in the neuroscience.

So far, some studies have suggested that N-GQDs presented low cytotoxicity and good biocompatibility [11, 12]. However, the inherent graphene-like properties of N-GQDs makes researchers be vigilant to their potential toxic effects in living bodies because the nanotoxicity of graphene have been extensively assessed during the past decade [13–15]. Recently, some researchers found the oxidative stress damages caused by N-GQDs, such as disrupting redox-sensitive system in model animals [16], or generating excessive ROS production [17, 18]. The brain is susceptible to redox imbalance due to their rich unsaturated fatty acids, so N-GQDs exposure could result in severe brain damage. Our previous study has demonstrated the induction of ferroptosis was triggered by N-GQDs exposure in microglia that are resident immune cells in the CNS [19]. Since ferroptosis has been defined as a critical form of cell death in neurodegenerative disorders following with iron overload and lipid peroxidation [20, 21], it seems that the GQDs-induced microglia death through ferroptosis pathway could increase risks on neuronal disorders in the CNS, but the underlying mechanisms remains unclear.

The necrotic cell death has been represented a known trigger of the immune system and resulted in inflammation in several organ injury, but the links between inflammation and ferroptosis has not been confirmed. An increasing number of studies have demonstrated the positive role of ferroptosis in inflammation through releasing damage-associated with molecular patterns (DAMPs) [22]. A half decade ago, researchers have found that GQDs induce inflammatory response following with two types of cell death, i.e. apoptosis and autophagy, in macrophages [23]. However, it is still unknown whether inflammatory reaction occurred in microglia ferroptosis caused by GQDs. Therefore, in this study, the underlying mechanisms of N-GQDs inducing ferroptosis and inflammation in microglia were thoroughly assessed.

Several studies have suggested that the intranasal administration made a drug be able to reach the brain via olfactory bulb, which indicated the valuable of nasal delivery of nanomedicines for brain disorders [7, 9]. Therefore, the intranasal administration used in this study not only investigated the neurotoxic effects of inhaled N-GQDs in surrounding environment, but also assessed the potential adverse effects of using N-GQDs as nasal delivery system in the diagnosis and treatment of brain disorders. The exposure dosages are based on the neuroscientific applied dosages of N-GQDs in several laboratory experiments, because this novel kind of nanoparticle has not yet been licensed in clinic applications [24–26]. In this study, the ferroptosis and inflammation induced by N-GQDs were observed in both hippocampus of mice and microglial BV2 cells. According to the data provided by a microarray analysis [27, 28] and available studies reporting the involvement of calcium-associated processes in the mechanisms of ferroptosis occurrence [29–31], we found that the disruption of calcium homeostatic levels through altering the normal opening of calcium channels in plasma and endoplasmic reticulum (ER) membranes could promote iron accumulation and ER stress response, and resulted in ferroptosis and inflammation in microglia after exposing to N-GQDs.

## Results

### Physiochemical properties of N-GQDs

**Fig. 1:**
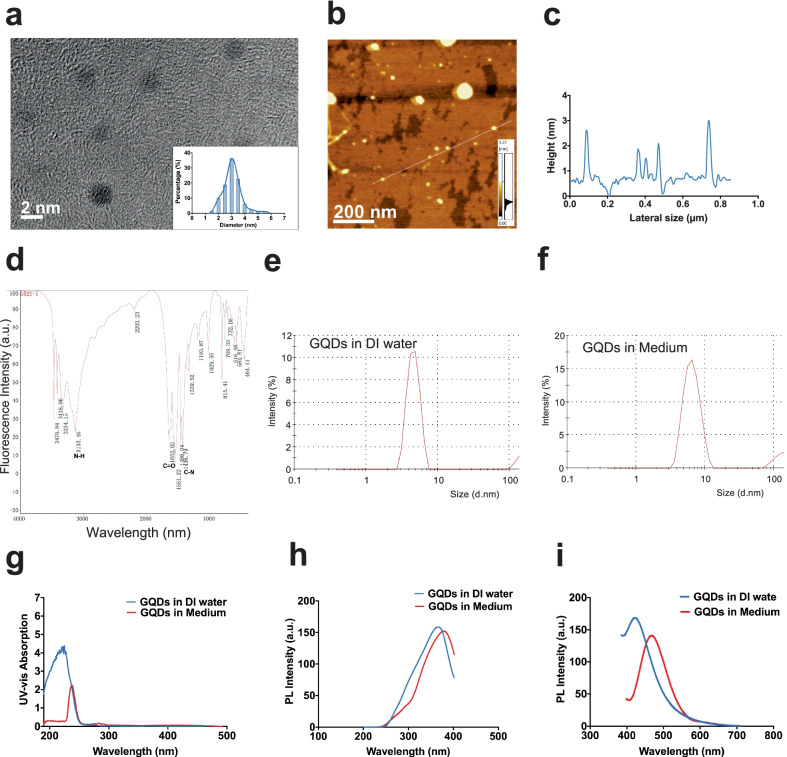
The characterization of N-GQDs. **a** The TEM images and size distribution of N-GQDs; **b**, **c** the AFM topography and height distribution of N-GQDs; **d** the FT-IR spectrum of N-GQDs; **e**, **f** the hydrous particle diameter of N-GQDs in deionized (DI) water and culture medium; **g** the UV–Vis absorption spectra of N-GQDs in DI water and culture medium; **h**, **i** the excitation and emission spectra of N-GQDs in DI water and culture medium at λem = 420 nm and 480 nm, or λex = 350 nm and 380 nm

According to TEM analysis, the average particle size of N-GQDs was uniformed and approximately 3 nm (Fig. 1a). The AFM topography of N-GQDs indicated the thickness of individual N-GQD from 0.5 to 3 nm (Fig. 1b, c). As the height of a classical graphene was around 0.35 nm, AFM data indicated that the layers of N-GQDs range from 1 to 4. The functional groups on the surface of N-GQDs were detected using a FI-IR spectrometry (Fig. 1d). The absorption peak at 3133 cm^−1^ was attributed to the stretching vibration of N–H bounds. The absorption peak at 1652 cm^−1^ was assigned to the stretching vibration of C=O bonds. The absorption peak at 1436 cm^−1^ was attributed to the stretching vibrations of C–N bonds, which indicated nitrogen doping to the GQDs.

Meanwhile, the properties of N-GQDs in deionized (DI) water and culture medium were assessed to keep up with the experimental environment. The hydrodynamic dimeters of 100 µg/mL N-GQDs in DI water and culture medium were similar but both slightly larger than TEM size (Fig. 1e, f). The UV–Vis absorption peaks of N-GQDs in two diluting solutions were in the blue spectral region, but the emission shifted to red from DI water to culture medium (Fig. 1g). When assessing their photoluminescence (PL), N-GQDs presented obvious fluorescence spectra, and their emission and excitation peaks in DI water centered at the wavelengthes slightly smaller than that in culture medium, while the fluorescent intensity in water was slightly stronger than that in culture medium (Fig. 1h, i). Since inflammatory responses to N-GQDs were investigated in this study, the endotoxin in the 100 µg/mL N-GQDs solution was firstly measured. The result was 0.0025 EU/mL that was much less than the limit of detection 0.01 EU/mL and could be considered as endotoxin free (Additional file 1: Fig. S1). The photoluminescence quantum yield (PLQY) of N-GQDs was both approximately 20% in the quality reports from manufacture (Additional file 1: Table S1).

### N-GQDs intranasal exposure caused ferroptosis, microglial activation and inflammation in hippocampus of mice

**Fig. 2:**
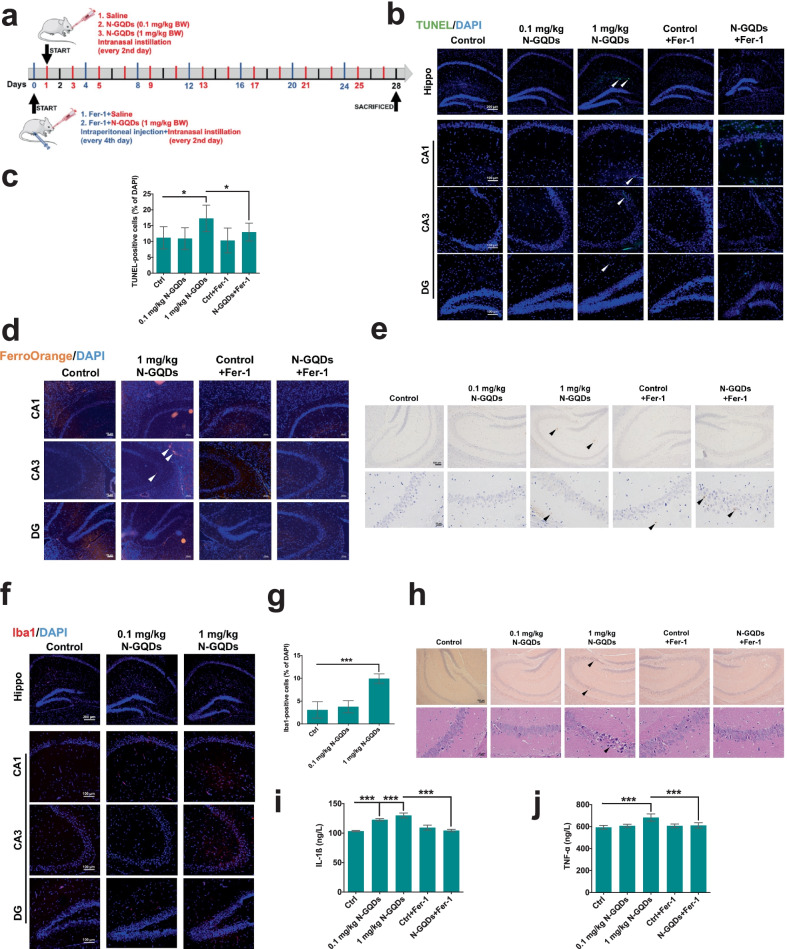
Fer-1 inhibited ferroptosis and neuroinflammation caused by intranasal N-GQDs exposure in the hippocampus. **a** Schematic workflow of the N-GQDs and Fer-1 treatments in mice; **b**, **c** representative fluorescence images presented dead cells showing green marked by TUNEL dye in hippocampus. The nucleus showed blue using DAPI. Arrows indicated TUNEL-positive cells. And the quantitative results of TUNEL positive cells; **d** representative fluorescence images presented ferrous iron level showing orange marked by FerroOrange dye in hippocampus. The nucleus showed blue using DAPI. Arrows indicated the ferrous iron accumulation; **e** representative immunohistochemical images presented 4-HNE in hippocampus. The arrows indicate increased expression of 4-HNE that displayed tan color; **f**, **g** representative immunofluorescence images presented microglia in hippocampus. The Iba1 showed red using primary antibodies with cy3-linked secondary antibodies, and the nucleus showed blue using DAPI. And the quantitative results of Iba1 positive cells; **h** representative histological images presented the hippocampus stained with H&E. The arrows indicated infiltrated inflammatory cells; **i**, **j** the levels of IL-1ß and TNF-α in hippocampus. For merely N-GQDs exposure groups, each mouse was intranasally instilled with saline, 0.1 and 1 mg/kg BW N-GQDs every other day for 28 days. For Fer-1 pre-treatment groups, each mouse was intraperitoneally injected with 5 mg/kg BW Fer-1 every fourth day and intranasally instilled with 1 mg/kg BW N-GQDs every other day for 28 days (mouse n = 6). Data are showed as mean ± SD of three independent experiments. The one-way ANOVA followed by the Dunnett’s t test were used to determine statistical significance (*P < 0.05, **P < 0.01, ***P < 0.001 vs. the control or 1 mg/kg BW N-GQDs)

Owning to the fluorescent property of N-GQDs, the distribution and accumulation of N-GQDs in the brain of mice treated with N-GQDs through nasal cavity were measured as the fluorescence intensity using an in vivo fluorescence imaging system for small animals. Fluorescence intensities in different areas of brain tissue exposed to N-GQDs exhibited stronger than the control (Additional file 1: Fig. S2). Generally, no severely side-effects, like mice death, occurred during the whole experiment (Fig. 2a), while slight changes in routine blood indicators (Additional file 1: Table S2), body weight and levels of two pro-inflammatory cytokines, i.e. IL-1ß and TNF-α, in serum (Additional file 1: Fig. S3) were observed in mice treated with N-GQDs. However, N-GQDs intranasal exposure damaged the hippocampus of mice when the exposure dose reached to 1 mg/kg BW N-GQDs, which was reflected by significantly reduction in brain coefficient and damaged morphology of neurons and glia (Additional file 1: Fig. S4a and S4b). Interestingly, these slightly impairments of hippocampus caused by N-GQDs could be alleviated by the ferroptosis specific inhibitor Fer-1.

After that, the high-dosed N-GQDs exposure significantly increased the number of dead cells that could be reversed by Fer-1 treatment (Fig. 2b, c), which indicated N-GQDs exposure could cause ferroptosis in hippocampus. Meanwhile, two typical ferroptosis characteristics that are iron overload, particularly ferrous iron accumulation (Fig. 2d, Additional file 1: Fig. S4c and S4d) and lipid peroxidation evidenced by increases in 4-HNE protein expression (Fig. 2e and Additional file 1: Fig. S5a–S5b), NADP + /NADPH ratio (Additional file 1: Fig. S5c) and MDA content (Additional file 1: Fig. S5d) were both observed in hippocampus of mice treated with N-GQDs, and the treatment of Fer-1 was capable of alleviating them as well.

In addition, microglial activation was detected by increased expression of specific protein Iba1 in hippocampus of mice treated with N-GQDs (Fig. 2f, g and Additional file 1: Fig. S4e). Meanwhile, the infiltration of inflammatory cells and increased levels of IL-1ß and TNF-α were observed in hippocampus of mice treated with N-GQDs in different degrees (Fig. 2h–j). That Fer-1 treatment inhibited microglial activation and inflammatory cytokines secretion (Additional file 1: Fig. S4f and Fig. 2h–j) indicated the close association between ferroptosis and neuroinflammation in hippocampus exposed to N-GQDs. Since activated microglia play an important role in the occurrence of neuroinflammation [32] and microglia were more sensitive to the toxicity of carbon-based nanomaterials than neurons [33], we speculated that N-GQDs might mainly trigger ferroptosis in microglia and results in neuroinflammation in the hippocampus, so the molecular mechanisms of N-GQDs causing ferroptosis were investigated in cultured microglial BV2 cells.

### N-GQDs induced ferroptosis and inflammatory cytokines release in microglia

**Fig. 3:**
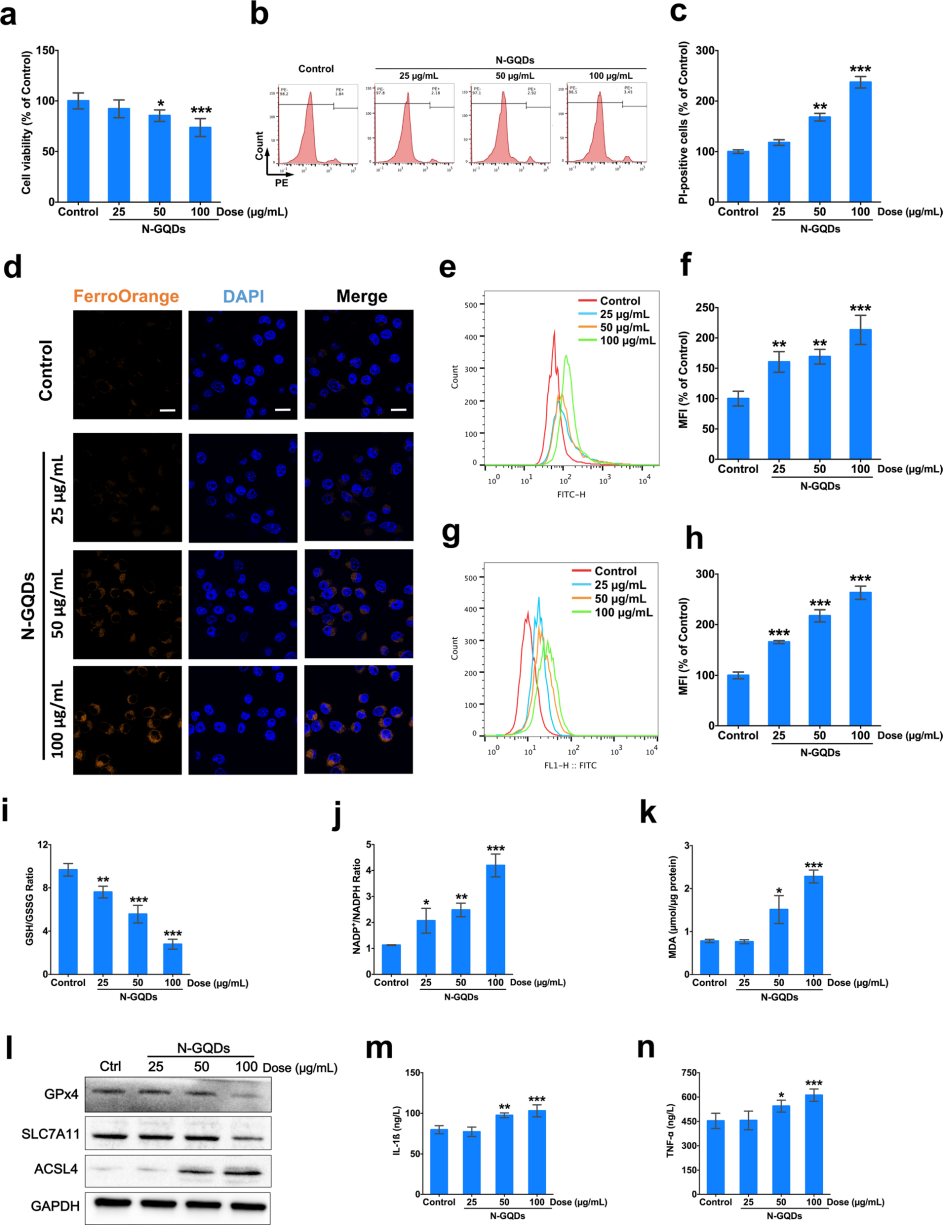
N-GQDs induced iron overload, lipid peroxidation and inflammation in BV2 cells at a dose-dependent manner. **a** The cell viability of BV2 cells; **b**, **c** representative PE fluorescence histogram plots of BV2 cells showed necrotic cells using PI dye, and the quantitative results of necrotic percentages; **d** representative fluorescent images of intracellular ferrous iron level in BV2 cells showed orange using FerroOrange dye. The nucleus showed blue using DAPI. Scale bar: 20 µm; **e**, **f** representative FITC fluorescence histogram plot of BV2 cells showed cytosolic ROS production using DCFH-DA dye, and the quantitative results of mean fluorescence intensity (MFI); **g**, **h** representative FITC fluorescence histogram plot of BV2 cells showed lipid ROS using C11BODIPY581/591 dye, and quantitative results of mean fluorescence intensity (MFI); **i**, **j**, **k** the GSH/GSSG ratio, NADP+/NADPH ratio and MDA content in BV2 cells; **l** the expressions of ferroptosis biomarker proteins SLC7A11, GPX4 and ACSL4 in BV2 cells using western blotting analysis; **m**, **n** the levels of IL-1ß, TNF-α and IL-6 in BV2 cells. BV2 cells were treated with 25, 50 and 100 μg/mL N-GQDs for 24 h (n = 3). Data are showed as mean ± SD of three independent experiments. The one-way ANOVA followed by the Dunnett’s t test were used to determine statistical significance (*P < 0.05, **P < 0.01, ***P < 0.001 vs. the control)

In the in vitro experimental environment, N-GQDs exposure at doses over 50 µg/mL for 24 h not only significantly decreased the cell viability of BV2 cells (Fig. 3a), but also increased the number of necrotic cells (Fig. 3b, c) at a dose-dependent manner when compared to the control. Moreover, typical characteristics of ferroptosis, including ferrous iron accumulation and lipid peroxidation, were measured in BV2 cells exposed to N-GQDs. The cytosolic ferrous iron levels that are identified by FerroOrange probes obviously increased when the treatment doses of N-GQDs were increasing in BV2 cells (Fig. 3d). Meantime, generations of cytosolic ROS and lipid ROS both enhanced in BV2 cells as the exposure concentrations of N-GQDs were enhancing (Fig. 3e–h). Moreover, the ratio of GSH and GSSG significantly decreased, while NADP^+^/NADPH and the end-product of lipid peroxidation, i.e. MDA content both increased when BV2 cells were exposed to N-GQDs with doses ranging from 25 to 100 µg/mL for 24 h (Fig. 3i–k). Tripeptide GSH serves as a protective role on antioxidant defence and NADPH is an important coenzyme of GSH reductase participating in maintaining the GSH content [34]. Therefore, GSH depletion and enhanced intracellular MDA level indicated N-GQDs disrupt the redox homeostasis and lead to lipid peroxidation in BV2 cells. The changed protein expressions of three widely-reported biomarkers of ferroptosis, i.e. proteins GPx4, SLC7A11 and ACSL4, were obviously observed in BV2 cells exposed to N-GQDs, especially 100 µg/mL N-GQDs. The expressions of GPx4 and SLC7A11 remarkably decreased while that of ACSL4 increased (Fig. 3l). The contents of IL-1ß and TNF-α were significantly higher in culture medium of BV2 cells exposed to N-GQDs at doses above 50 µg/mL than the control, which determined the inflammatory reaction to the N-GQDs (Fig. 3m, n).

**Fig. 4:**
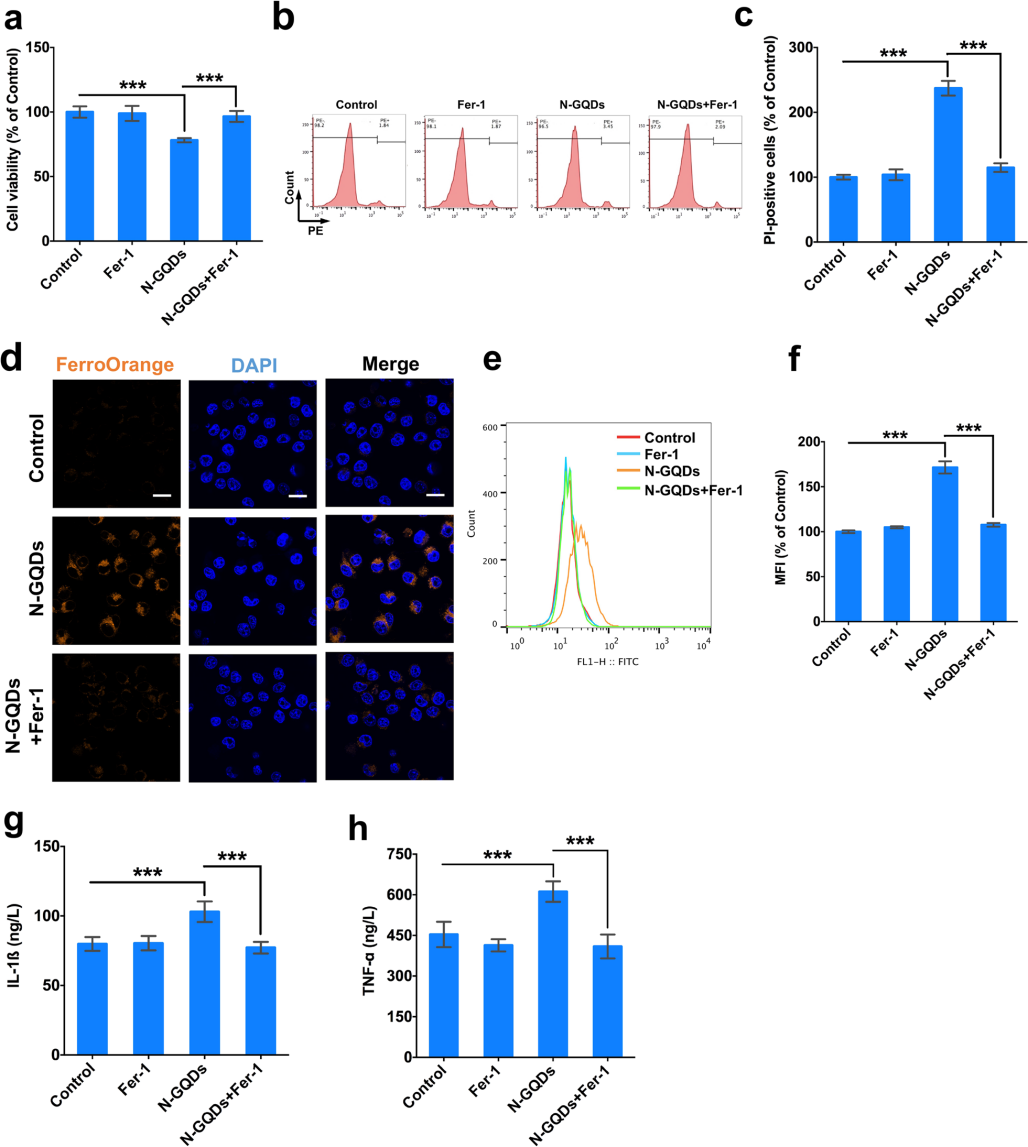
N-GQDs caused ferroptosis following with inflammatory cytokines release in BV2 cells. **a** The cell viability of BV2 cells; **b**, **c** representative PE fluorescence histogram plots of BV2 cells showed necrotic cells using PI dye, and the quantitative results of necrotic percentages; **d** representative fluorescent images of intracellular ferrous iron level in BV2 cells showed orange using FerroOrange dye. The nucleus showed blue using DAPI. Scale bar: 20 µm; **e**, **f** representative FITC fluorescence histogram plot of BV2 cells showed lipid ROS using C11BODIPY581/591 dye, and the quantitative results of mean fluorescence intensity (MFI); **g**, **h** the levels of IL-1ß, TNF-α and IL-6 in BV2 cells. BV2 cells were pre-treated with Fer-1 for 2 h and then exposed to 100 µg/mL N-GQDs for 24 h (n = 3). Data are showed as mean ± SD of three independent experiments. The one-way ANOVA followed by the Dunnett’s t test were used to determine statistical significance (*P < 0.05, **P < 0.01, ***P < 0.001 vs. the control or 100 µg/mL N-GQDs)

After detecting ferrous iron overload and lipid peroxidation, the pre-treatment of a ferroptosis inhibitor Fer-1 was used to confirm whether N-GQDs exposure was capable of triggering ferroptosis in microglia. Since Fer-1 was reported to effectively inhibit lipid peroxidation in order to protect cells against ferroptosis, increased lipid ROS generation, decreased GSH/GSSG ratio and enhanced MDA content in BV2 cells treated with N-GQDs were all found to be all relieved by Fer-1 pre-treatment (Additional file 1: Fig. S6). Meanwhile, the data suggested that Fer-1 pre-treatment not only significantly inhibited the decreased cell viability and increased necrosis (Fig. 4a–c), but also alleviated the intracellular iron accumulation and excessive ROS production (Fig. 4d–f) in BV2 cells treated with N-GQDs. Furthermore, Fer-1 was capable of notably reversing the excessive secretions of pro-inflammatory cytokines IL-1ß and TNF-α from BV2 cells treated with 100 µg/mL N-GQDs (Fig. 4g, h).

### Intracellular calcium overload caused by N-GQDs through activating calcium channels of L-VGCCs and RyR triggered ferroptosis in microglia

**Fig. 5:**
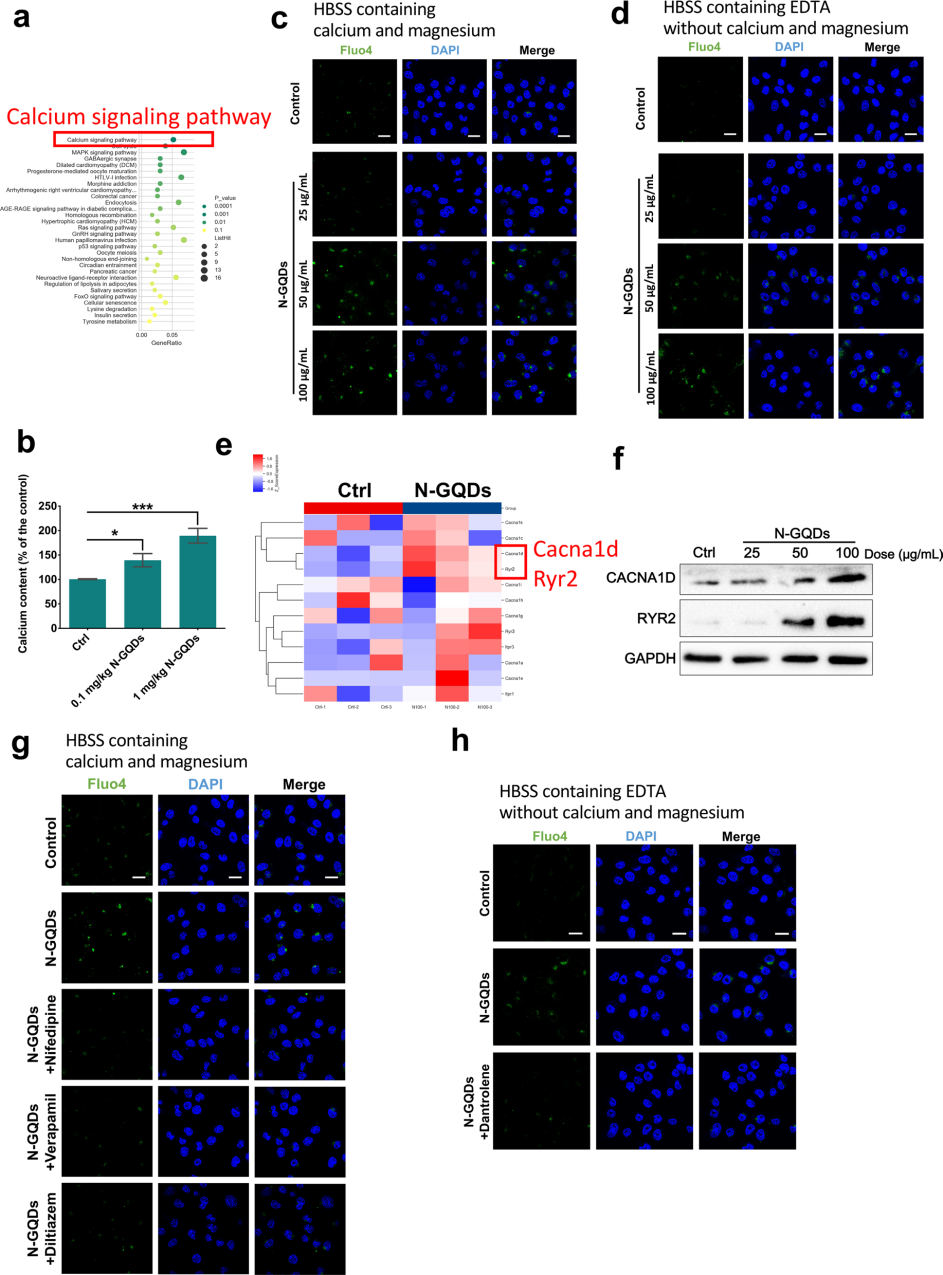
N-GQDs increased intracllular calcium level through activating calcium channels of L-VGCC and RyR in BV2 cells. **a** KEGG signaling pathways enriched by differentially expressed genes caused by 100 µg/mL N-GQDs in BV2 cells for 24 h using microarray analysis; **b** the levels of calcium in hippocampus; **c**, **d** representative fluorescent images of intracellular calcium level in BV2 cells showed green using Fluo4 dye with HBSS containing calcium and magnesium or HBSS containing EDTA without calcium and magnesium. The nucleus showed blue using DAPI, Scale bar: 20 µm; **e** heatmap of differentially expressed genes associated with calcium signaling pathway in BV2 cells treated with 100 µg/mL N-GQDs for 24 h using microarray analysis; **f** the protein expressions of CACNA1D and RYR2 in BV2 cells using western blotting analysis; **g**, **h** representative fluorescent images of intracellular calcium level in BV2 cells showed green using Fluo4 dye with HBSS containing calcium and magnesium or HBSS containing EDTA without calcium and magnesium. The nucleus showed blue using DAPI, Scale bar: 20 µm. Each mouse was intranasally instilled with saline, 0.1 and 1 mg/kg BW N-GQDs every other day for 28 days (mouse n = 6). BV2 cells were treated with 25, 50 and 100 μg/mL N-GQDs for 24 h or BV2 cells were pre-treated with nifedipine, diltiazem, verapamil and dantrolene for 2 h and then exposed to 100 µg/mL N-GQDs for 24 h (n = 3). Data are showed as mean + SD of three independent experiments. Data are showed as mean ± SD of three independent experiments. The one-way ANOVA followed by the Dunnett’s t test were used to determine statistical significance (*P < 0.05, **P < 0.01, ***P < 0.001 vs. the control)

That microarray data indicated genes altered by N-GQDs were mostly enriched in calcium signaling pathway suggested that intracellular calcium homeostasis imbalance was involved in N-GQDs damaging microglia (Fig. 5a). After the significantly increased calcium contents in hippocampus of mice treated with N-GQDs were observed (Fig. 5b), the alternations of cytosolic calcium levels in BV2 cells treated with N-GQDs were assessed as well. As we know, there are two sources of cytosolic calcium in cells. One is calcium ions flowing from cellular outside to the cytoplasm, and the other is calcium ions flowing from intracellular calcium stores (organelles of ER and mitochondria) to cytoplasm [35]. Under the circumstances of extracellular environment with/without calcium and magnesium, the cytosolic calcium levels detected by Fluo-4 probe were all increasing along with the increasing administration doses of N-GQDs in BV2 cells (Fig. 5c, d), which suggested that N-GQDs enhanced the intracellular calcium level through the two main ways of cytosolic calcium influx. According to the microarray data, we found that the expressions of genes coding L-type voltage-gated calcium channels (L-VGCCs) in plasma membrane and ryanodine receptor (RyR) calcium channels, especially genes cacna1d and ryr2, significantly increased after exposing to 100 µg/mL N-GQDs in BV2 cells (Fig. 5e). The western blotting analysis also confirmed that N-GQDs exposure remarkably increased the expressed levels of protein CACNA1D and RYR2 at a dose-dependent manner in BV2 cells (Fig. 5f). Meanwhile, three types of L-VGCCs inhibitors, i.e. nifedipine, diltiazem and verapamil, and a RyR channels inhibitor dantrolene all reversed the cytosolic calcium levels increased by N-GQDs in BV2 cells (Fig. 5g, h), which indicated that N-GQDs could activate the open of L-VGCCs and RyR channels and resulted in intracellular calcium overload.

**Fig. 6:**
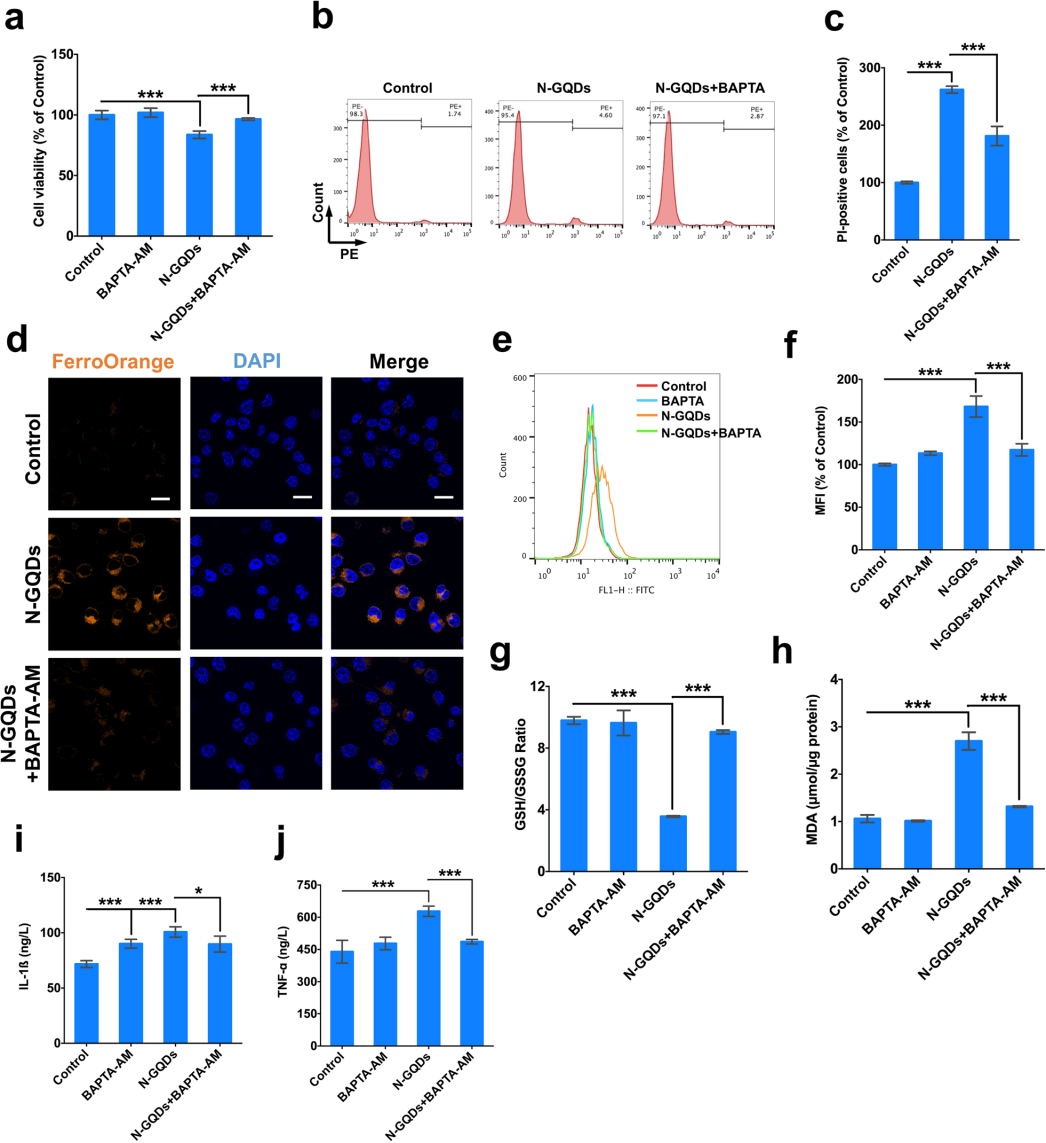
The intracellular calcium imhomeostasis caused by N-GQDs triggered ferroptosis in BV2 cells. **a** The cell viability of BV2 cells; **b**, **c** representative PE fluorescence histogram plots of BV2 cells showed necrotic cells using PI dye, and the quantitative results of necrotic percentages; **d** representative fluorescent images of intracellular ferrous iron level in BV2 cells showed orange using FerroOrange dye. The nucleus showed blue using DAPI, Scale bar: 20 µm; **e**, **f** representative FITC fluorescence histogram plot of BV2 cells showed lipid ROS using C11BODIPY581/591 dye, and the quantitative results of mean fluorescence intensity (MFI) from flow cytometer analysis; **g**, **h** the GSH/GSSG ratio and MDA content in BV2 cells; **i**, **j** the levels of IL-1ß and TNF-α in BV2 cells. BV2 cells were pre-treated with BAPTA-AM for 2 h and then exposed to 100 µg/mL N-GQDs for 24 h (n = 3). Data are showed as mean ± SD of three independent experiments. The one-way ANOVA followed by the Dunnett’s t test were used to determine statistical significance (*P < 0.05, **P < 0.01, ***P < 0.001 vs. the control or 100 µg/mL N-GQDs)

When the pre-treatment of a membrane permeable calcium chelator BAPTA-AM was capable of alleviating the cytosolic calcium overload caused by N-GQDs (Additional file 1: Fig. S7), it also significantly reversed the cell viability damage and increased necrotic cells induced by N-GQDs (Fig. 6a–c). The enhanced intracellular ferrous iron content (Fig. 6d), lipid ROS generation (Fig. 6e, f), decreased GSH/GSSG ratio (Fig. 6g) and increased MDA level (Fig. 6h) were all relived by BAPAT-AM pre-treatment, which suggested that calcium imbalance might be involved in ferroptosis triggered by N-GQDs in microglia. In addition, the excessive releases of inflammatory cytokines in N-GQDs-treated cells were all alleviated by BAPTA-AM to some extent (Fig. 6i, j).

### L-VGCCs were involved in calcium-iron crosstalk to increase intracellular iron level and trigger ferroptosis in N-GQDs-treated microglia

**Fig. 7:**
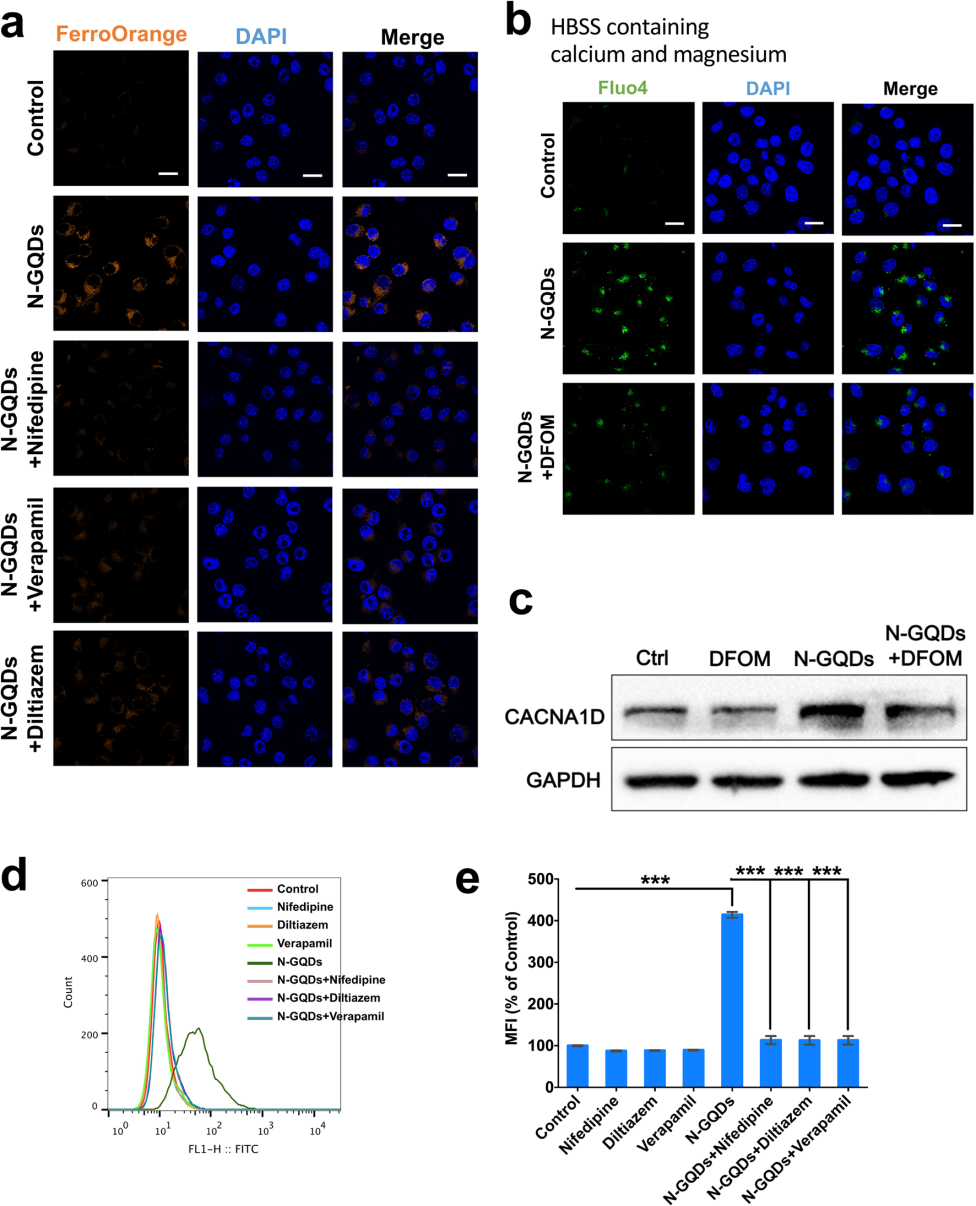
N-GQDs induced iron overload through calcium-iron crosstalk mediated by L-VGCCs in BV2 cells. **a** Representative fluorescent images of intracellular ferrous iron level in BV2 cells showed orange using FerroOrange dye. The nucleus showed blue using DAPI, Scale bar: 20 µm; **b** representative fluorescent images of intracellular calcium level in BV2 cells showed green using Fluo4 dye with HBSS containing calcium and magnesium. The nucleus showed blue using DAPI, Scale bar: 20 µm; **c** the protein expression of CACNA1D in BV2 cells using western blotting analysis; **d**, **e** representative FITC fluorescence histogram plot of BV2 cells showed cytosolic ROS production using DCFH-DA dye, and the quantitative results of mean fluorescence intensity (MFI). BV2 cells were pre-treated with nifedipine, diltiazem, verapamil and DFOM for 2 h and then exposed to 100 µg/mL N-GQDs for 24 h (n = 3). Data are showed as mean ± SD of three independent experiments. The one-way ANOVA followed by the Dunnett’s t test were used to determine statistical significance (*P < 0.05, **P < 0.01, ***P < 0.001 vs. the control or 100 µg/mL N-GQDs)

After confirming the inhibitory effects of L-VGCCs inhibitors nifedipine, diltiazem and verapamil on the expression of CACNA1D in BV2 cells (Additional file 1: Fig. S8a), the reduction in cell viability (Additional file 1: Fig. S8b–S8d) as well as the enhancements in necrosis (Additional file 1: Fig. S8e and S8f) and inflammatory cytokines release (Additional file 1: Fig. S8g and S8h) were all reversed to some extent by these inhibitors in N-GQDs-treated BV2 cells. When investigating the role of L-VGCCs in the ferroptosis, L-VGCCs inhibitors were found to alleviate the intracellular ferrous iron accumulation (Fig. 7a), while an iron chelator DFOM alleviated the intracellular calcium overload and increased expression of CACNA1D (Fig. 7b, c) in BV2 cells treated with N-GQDs. The findings indicated that calcium-iron crosstalk mediated by L-VGCCs might be involved in the iron overload caused by N-GQDs in BV2 cells. Additionally, L-VGCCs inhibitors reversed the redox imbalance evidenced by excessive productions of cytosolic ROS (Fig. 7d, e) and lipid ROS (Additional file 1: Fig. S9a and S9b), decreased GSH/GSSG ratio (Additional file 1: Fig. S9c) and increased MDA content (Additional file 1: Fig. S9d) in BV2 cells after exposing to 100 µg/mL N-GQDs.

### RyR channels were involved in ER stress response induced by calcium imhomeostasis to trigger ferroptosis in N-GQDs-treated microglia

**Fig. 8:**
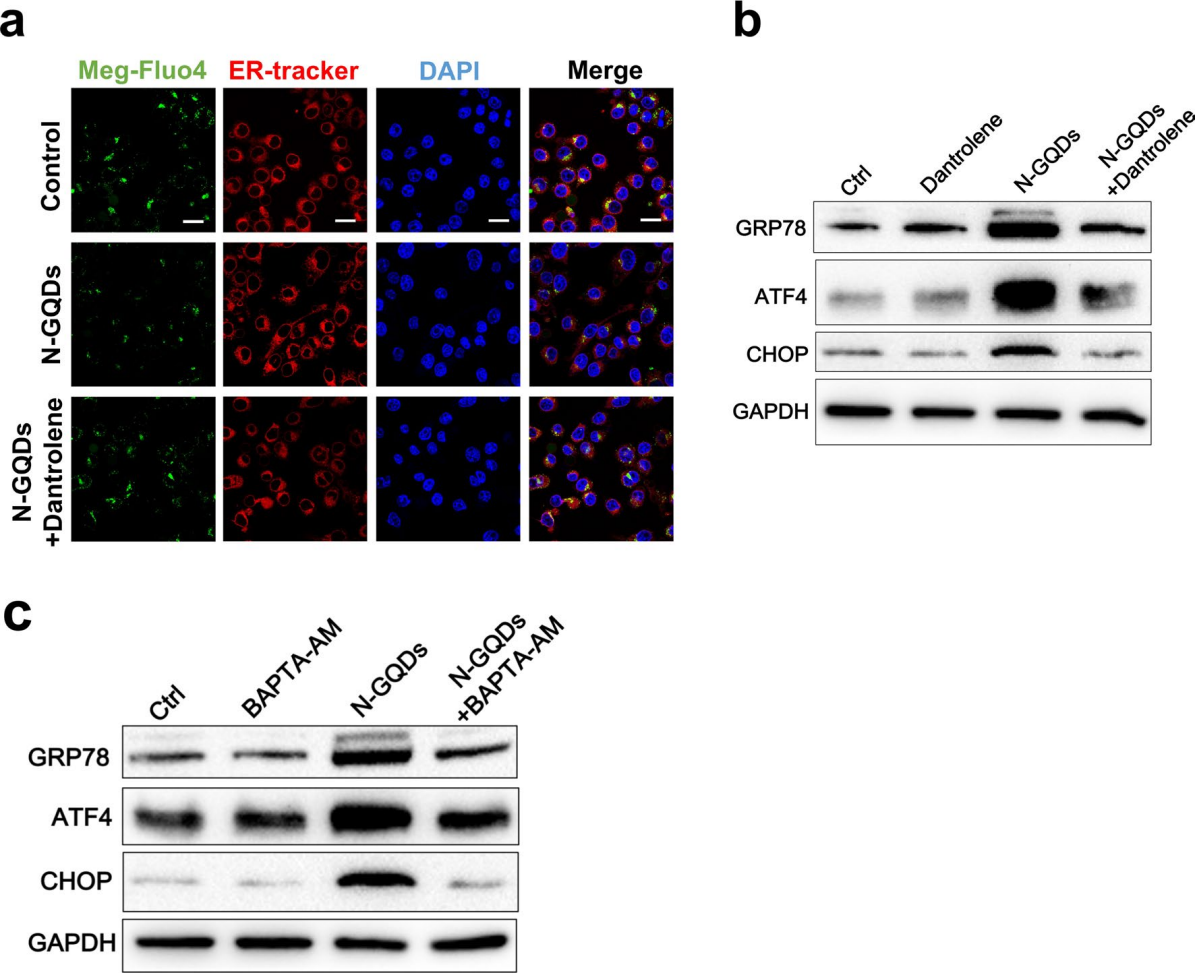
ER stress response to N-GQDs was caused by RyR channel mediated calcium imhomeostasis in BV2 cells. **a** Representative fluorescent images of ER calcium level in BV2 cells showed green using meg-Fluo4 dye with HBSS containing EDTA without calcium and magnesium. The ER showed red using ER-Tracker Red, and the nucleus showed blue using DAPI, Scale bar: 20 µm; **b**, **c** the protein expressions of GRP78, CHOP and ATF4 in BV2 cells using western blotting analysis. BV2 cells were pre-treated with dantrolene and BAPTA-AM for 2 h and then exposed to 100 µg/mL N-GQDs for 24 h (n = 3). Data are showed as mean ± SD of three independent experiments. The one-way ANOVA followed by the Dunnett’s t test were used to determine statistical significance (*P < 0.05, **P < 0.01, ***P < 0.001 vs. the control or 100 µg/mL N-GQDs)

After confirming the RyR channels inhibitor dantrolene reversed the enhanced RyR2 protein expression (Additional file 1: Fig. S10a) caused by N-GQDs in BV2 cells, the inhibitor also reversed reduced cell viability (Additional file 1: Fig. S10b), enhanced necrotic cells (Additional file 1: Fig. S10c and S10d) and excessive inflammatory cytokines release (Additional file 1: Fig. S10e and S10f). The lipid peroxidation in BV2 cells treated with N-GQDs were alleviated by the RyR channels inhibitor as well (Additional file 1: Fig. S11). When assessing the role of RyR channels in ferroptosis, we notice the mediation of RyR channels in the normal function of ER. When N-GQDs-exposed BV2 cells were pre-treated with RyR channels inhibitor, the decreases in the calcium level in ER detected by meg-Fluo4 probes and increases in the protein expressions of ER stress biomarkers were remarkably reversed (Fig. 8a, b). Meanwhile, calcium chelator BAPTA-AM was capable of alleviating the enhanced expressions of ER stress proteins induced by N-GQDs (Fig. 8c), which indicated that calcium imhomeostasis mediated by RyR channels caused ER stress in BV2 cells exposed to N-GQDs.

ER stress were then thoroughly assessed in BV2 cells treated with N-GQDs. TEM technique was used to observe ER and other critical organelles damaged by N-GQDs in hippocampus and BV2 cells. According to TEM images, ER and mitochondria were damaged by N-GQDs exposure in vivo and in vitro to some extent (Additional file 1: Fig. S12a and S12b). In high-dosed N-GQDs treatment groups, some ERs were swollen or dilated, while some mitochondria were shrinkage or had broken ridges. When the microarray data suggested genes associated with ER stress changed by N-GQDs in microglia (Additional file 1: Fig. S13a), the changed expressions of four typical ER stress relevant genes, i.e. ddit3, hspa5, igbp1 and atf4, were confirmed in BV2 cells exposed to N-GQDs (Additional file 1: Fig. S13b–S13e). In the hippocampus of mice treated with N-GQDs, we observed not only the higher expressed GRP78 in neuronal cells than the control (Additional file 1: Fig. S12c), but also the increased expression levels of ER stress biomarkers, i.e. protein GRP78, CHOP and ATF4 (Additional file 1: Fig. S12d). Meantime, the expression levels of proteins GRP78, ATF4 and CHOP notably increased by N-GQDs in BV2 cells (Additional file 1: Fig. S12e), which might be related with the uptake of N-GQDs in ERs (Additional file 1: Fig. S14).

**Fig. 9:**
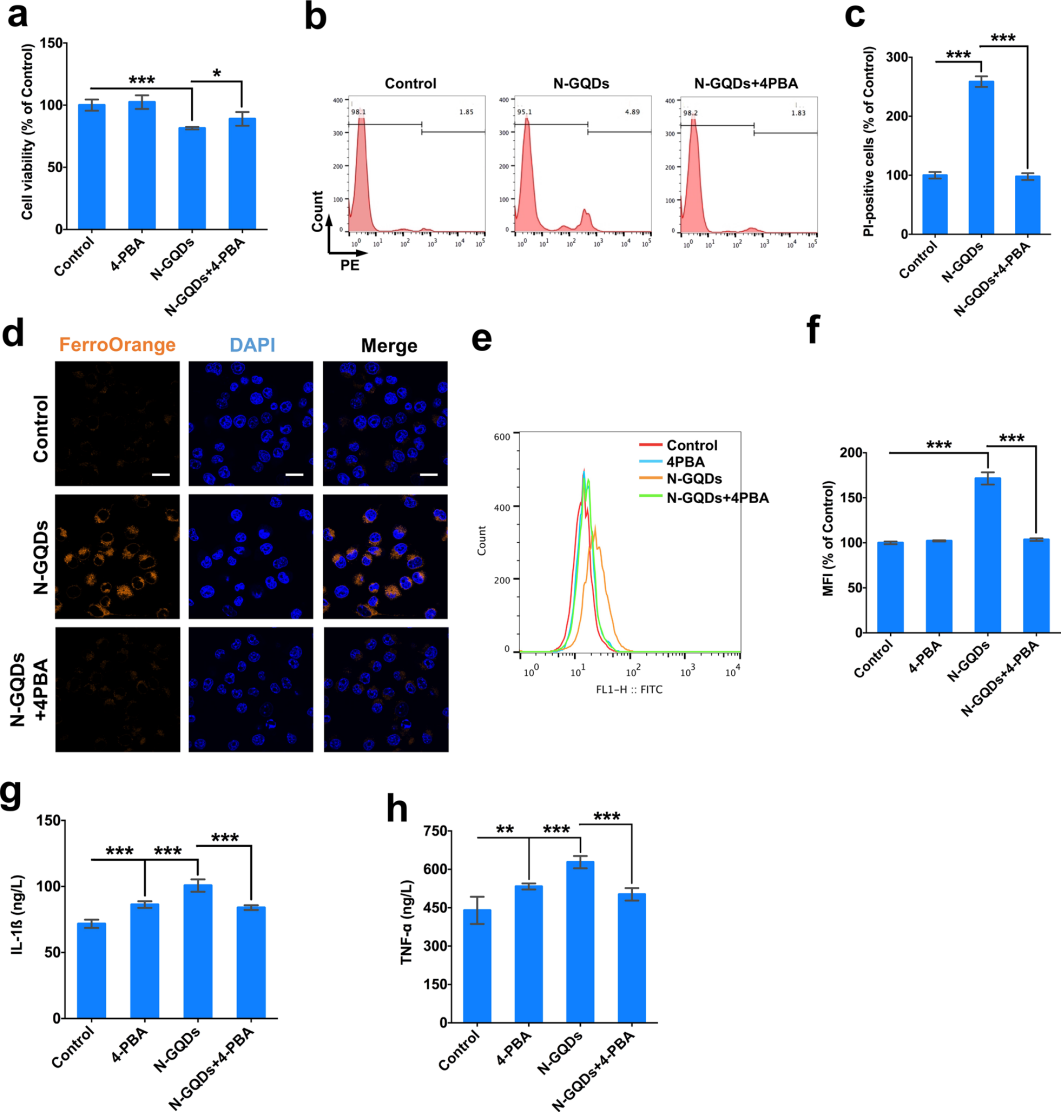
N-GQDs-caused ER stress triggered iron overload, lipid peroxidation and inflamamtion in BV2 cells. **a** The cell viability of BV2 cells; **b**, **c** representative PE fluorescence histogram plots of BV2 cells showed necrotic cells using PI dye, and the quantitative results of necrotic percentages from the flow cytometer analysis; **d** representative fluorescent images of intracellular ferrous iron level in BV2 cells showed orange using FerroOrange dye. The nucleus showed blue using DAPI, Scale bar: 20 µm; **e**, **f** representative FITC fluorescence histogram plot of BV2 cells showed lipid ROS using C11BODIPY581/591 dye, and the quantitative results of mean fluorescence intensity (MFI); **g**, **h** the levels of IL-1ß and TNF-α in BV2 cells. BV2 cells were pre-treated with 4-PBA for 2 h and then exposed to 100 µg/mL N-GQDs for 24 h (n = 3). Data are showed as mean ± SD of three independent experiments. The one-way ANOVA followed by the Dunnett’s t test were used to determine statistical significance (*P < 0.05, **P < 0.01, ***P < 0.001 vs. the control or 100 µg/mL N-GQDs)

A specific ER stress inhibitor 4-PBA that was found to inhibit the increased expressions of ER stress proteins caused by N-GQDs (Additional file 1: Fig. S15) was used to assess whether ER stress response to N-GQDs was a reason to trigger ferroptosis in microglia. When the pre-treatment of 4-PBA was observed to effectively reverse the decreased cell viability and increased number of necrotic cells caused by N-GQDs in BV2 cells (Fig. 9a–c), the cytosolic ferrous iron accumulation and lipid ROS generation occurred in BV2 cells were inhibited by 4-PBA as well (Fig. 9d–f). Meanwhile, even though 4-PBA significantly increased secretions of inflammatory cytokines IL-1ß and TNF-α compared to the control, it was capable of alleviating excessively released inflammatory cytokines when compared to the merely N-GQDs-treatment group (Fig. 9g, h).

## Discussion

The increasing exposure of N-GQDs to the environment and public following with their wide applications has become an important public health problem. It has been reported that the administration from nasal cavity to brain through the retrograde transport along the olfactory nerve is a common route for carbon-based nanoparticles, including GQDs, which indicated the CNS could be an important toxic target to carbon-based nanoparticles [4, 36]. Moreover, along with the bright prospect on the application of N-GQDs in fields of biomedicine and neuroscience due to their good biocompatibility and excellent fluorescent properties [2, 7], it is critical to assess the biosafety of N-GQDs in the CNS before permitting their applications in the clinic. In this study, first of all, we assured intranasal exposed N-GQDs were capable of transporting into and accumulating in the brain of mice and hippocampus was one of main targets. Several studies have suggested that nanoparticles exposure via inhalation could be transported to the brain along the olfactory nerve with neurotoxic effects [5]. Although the relative surface area of the nasal olfactory mucosa in humans (5%) is much smaller than that of rodents (50%), the difference only makes humans less susceptible to airborne pollutants, but the neurotoxic effects would be similar [4]. The findings demonstrated some slight adverse effects in N-GQDs-deposited hippocampus, including cell death, microglial activation and neuroinflammation.

Recently, the ability of nanomaterials inducing different forms of regulated cell death, including ferroptosis, has been reported to benefit for cancer therapy [37, 38]. However, the ferroptosis induced by nanomaterials and the consequences in healthy bodies should be assessed at the first place. Our findings suggested that a specific ferroptosis inhibitor Fer-1 was capable of not only reversing the cell death, iron overload and lipid peroxidation, but also alleviating the microglial activation, inflammatory cells infiltration and inflammatory cytokines releases in hippocampus of mice intranasally exposed to N-GQDs, which indicated N-GQDs exposure triggering ferroptosis in neuronal cells and the association between ferroptosis and neuroinflammation. As we know, microglia participate in the neuroinflammatory responses to several types of carbon-based nanomaterials and quantum dots [4, 33], and they are more sensitive to the toxicity of carbon-based nanomaterials than other kinds of neuronal cells [33]. Therefore, microglia might be the main toxic target of N-GQDs, and N-GQDs-triggered ferroptosis in microglia might contribute to the impairments and inflammation in hippocampus of mice. Based on this hypothesis, this study focused on investigating the toxic mechanisms of N-GQDs inducing microglia ferroptosis in the microglial BV2 cell line that is a convenient and stable cell line and wildly used in the fields of neurotoxicology and immunotoxicology [39].

Similar to our previous study [19], N-GQDs exposure was found to enhance the levels of intracellular ferrous iron and disrupt redox disequilibrium when they decreased the cell viability and increased the quantity of necrotic cell at a dose-dependent manner in BV2 cells. Fer-1 was used to confirm the ferroptosis induced by N-GQDs in BV2 cells. In this study, we further assessed the inflammation caused N-GQDs and their relationship with ferroptosis, because ferroptosis has been reported to cause DAMPs release that leads to the recruitment of inflammatory cells and inflammation injury [21, 22]. Except that Fer-1 attenuated the neuroinflammatory effects caused by N-GQDs in hippocampus, the ferroptosis inhibitor was capable of reversing the secretion of inflammatory cytokines from BV2 cells, which indicated the close association between ferroptosis and inflammation that were both induced by N-GQDs treatment.

The microarray data suggested that calcium signaling pathways could take part in ferroptosis caused by N-GQDs in BV2 cells. Recently, there have been some studies reporting the role of intracellular calcium overload in the ferroptosis induced by different ferroptosis inducers [29–31]. A calcium-influx-dependent neuronal cell death, discovered almost three decades ago and eventually named oxytosis that involves GSH depletion, ROS generation and lipid peroxidation and shares many same steps of ferroptosis [40]. However, it is still unclear on the mechanisms of calcium imhomeostasis involvement in the ferroptosis process induced by nanomaterials. In BV2 cells, N-GQDs enhanced cytosolic calcium content through activating the L-VGCCs in plasma membrane to promote extracellular calcium influx and RyR channels in ER to promote calcium release from intracellular calcium pools. When eliminating the overloaded cytosolic calcium content in BV2 cells by a calcium chelator, the ferroptosis effects and excessive inflammatory cytokines secretion caused by N-GQDs were all inhibited, which indicated that N-GQDs might trigger ferroptosis and inflammation through disrupting the calcium homeostasis. Under this circumstance, the specific roles of L-VGCCs and RyR calcium channels were investigated in the calcium-dependent ferroptosis induced by N-GQDs.

Several studies have reported that excess free iron is capable of entering into cells through L-VGCCs and results in increased ROS generated via Fenton reactions [41–43]. When the L-VGCCs were inhibited by three types of specific inhibitors, the increased levels of ferrous iron in BV2 cells treated with N-GQDs was reversed, so were oxidation stress and lipid peroxidation. The findings suggested that N-GQDs might activate the opening of L-VGCCs in BV2 cells to promote influx of both calcium and iron from cellular outside. Moreover, many recent studies have demonstrated that the relationship between calcium and iron is Janus-faced, and iron overload was reported to induce the calcium contents above homeostatic levels via stimulating calcium signals [31, 44]. Therefore, an iron chelator was found to reverse the cytoplasmic calcium levels and CACNA1D expression both increased by N-GQDs in BV2 cells, which indicated that N-GQDs could cause intracellular iron overload through calcium-iron crosstalk mediated by L-VGCCs and lead to ferroptosis.

As mentioned above, the increased plasmatic calcium level was also attributed to the calcium release from the ER mediated by RyR calcium channels in BV2 cells exposed to N-GQDs. RyR channels were reported to be involved in ER stress responses via mediating cytosolic calcium-induced calcium release from ER to destroy ER calcium homeostasis [45–47]. In this study, we found the ER stress response to N-GQDs through inducing unfolded protein response (UPR) and subsequently activating the ATF4-CHOP pathway in hippocampus of mice and BV2 cells at a dose-dependent manner, so RyR channels could contribute to the ER stress through disrupting the cytosolic and ER calcium homeostasis.

ER stress has been reported to be involved in a variety of cellular signaling pathways that determine the fate of cell in large part, including ferroptosis and inflammation [48, 49]. Moreover, the critical role of ER stress in many other types of programmed cell death have been confirmed [50, 51]. With cells pre-treating with a typical inhibitor of ER stress, we found that ER stress might play an indispensable role in N-GQDs triggering ferroptosis and inflammatory reaction in BV2 cells. In summary, RyR channels activated by N-GQDs caused calcium imhomeostasis to induce ER stress, and then increased ROS production to trigger ferroptosis and inflammation in BV2 cells.

**Fig. 10:**
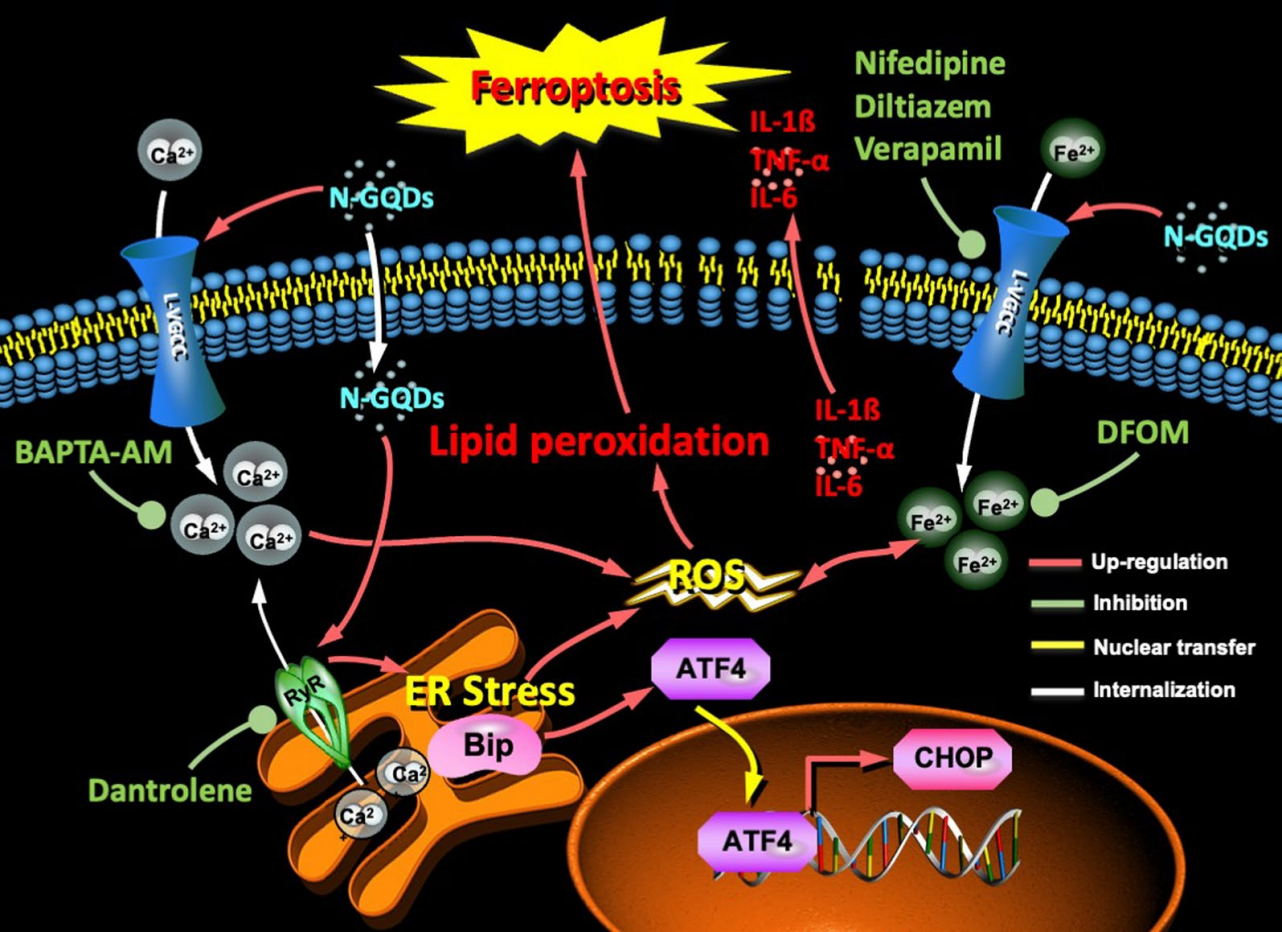
Proposed schematic diagram for N-GQDs-induced ferroptosis and inflammation in BV2 cells. ER stress: endoplasmic reticulum stress; ROS: reactive oxygen species; DFOM: deferoxamine mesylate

In this study, the findings indicated that N-GQDs disrupted intracellular calcium homeostasis by influencing two calcium channels, i.e. L-VGCCs and RyR channels, to promote deleterious calcium-iron crosstalk and ER stress response, which both increased ROS generation and resulted in ferroptosis and excessive inflammatory cytokines release in microglia treated with N-GQDs (Fig. 10). Although triggering ferroptosis via nanomaterials, like biocompatible GQDs, is a promising therapy for brain cancer [37], GQDs could cause health consequences in normal CNS because brains are vulnerable to lipid peroxidation. There is a growing body of evidence that ferroptosis plays a central role in the pathophysiology of several neurodegenerative diseases, including Alzheimer’s disease, Parkinson’s disease, and Huntington’s disease [52]. Thus, GQDs-caused ferroptosis in hippocampus might lead to loss of neuronal function, like learning and memory impairments that are similar to Alzheimer’s disease. Our next step should assess the cognitive impairments caused by N-GQDs in mammals through several behaviour tests in order to provide more information on the neurotoxicity of GQDs, which is capable of assuring their biosafe applications in humans, especially high-risk population to neurodegenerative disorders.

## Conclusion

In this study, intranasally exposed N-GQDs could be translocated into hippocampus of mice and caused cell death, iron overload and lipid peroxidation, which all could be impeded by a ferroptosis-specific inhibitor. Since microglial activation and neuroinflammation were observed in hippocampus treated with N-GQDs, microglia might be the main target of N-GQDs. Thus, the microglial BV2 cell line was used to investigate the molecular mechanisms of N-GQDs causing ferroptosis following with inflammatory reactions. According to the microarray data showing the calcium signaling pathway mostly influenced by N-GQDs exposure, we found that the increased cytosolic calcium levels mediated by two calcium channels, i.e. L-VGCCs and RyR channels, was involved in N-GQDs-triggered ferroptosis in BV2 cells. The opening of L-VGCCs in plasma membrane was found to contribute to the iron accumulation through free iron influx and calcium-iron crosstalk in BV2 cells exposed to N-GQDs. Meanwhile, N-GQDs-caused activation of RyR channels disrupted the calcium homeostasis in cytoplasm and ER to mediate ER stress that plays a role in triggering ferroptosis and inflammatory cytokines secretion in BV2 cells. This study not only indicated the mechanisms of deleterious calcium imhomeostasis in nanomaterials inducing ferroptosis, but also reminded researchers to pay more attention on the mechanism-based risk assessment of so called low-toxic nanoparticles.

## Methods

### Reagents

ToxinSensorTM Chromogenic LAL Endotoxicn Assay kit was purchased from GenScript (Nanjing, China). The GPx4, SLC7A11, ACSL4, CACNA1D, RYR2, CHOP, GRP78, ATF4 and GAPDH primary antibodies, and secondary antibodies of Cy3 conjugated anti-mouse IgG and the HRP conjugated anti-rabbit IgG were all purchased from ABclonal Technology (Wuhan, China). The 4-HNE primary antibody was purchased from Abcam (Cambs, UK). The IBA1 primary antibody was purchased from Cell Signaling Technology, Inc. C11-BODIPY^581/591^ probe was purchased from Thermo Fisher Scientific (MA, USA). FerroOrange probe and Iron Assay Kit with Colorimetric were purchased from Dojindo Molecular Technology (Japan). DAPI dye, 2′,7′-dichlorodihydrouorescein diacetate (DCFH-DA) probe, Fluo-4 AM probe, GSH/GSSG Assay Kit, NADP^+^/NADPH Assay Kit with WST-8, Lipid Peroxidation MDA Assay Kit and a calcium colorimetric assay kit were purchased from Beyotime Biotechnology (Shanghai, China). Cell Counting Kit 8 (CCK8), propidium iodide (PI), ER-Tracker Red and Fluo-4 AM were all purchased from Yeasen Biotechnology (Shanghai, China). Ferrostain-1 (Fer-1), BAPTA-AM, ethylenediaminetetraacetic acid trisodium salt (EDTA trisodium salt), nifedipine, diltiazem, verapamil, deferoxamine mesylate (DFOM) and dantrolene (sodium salt) were all purchased from APExBIO (TX, USA). IL-1ß, IL-6 and TNF-α commercial reagent kits were purchased from Yi Fei Xue Biotechnology (Nanjing, China). Mag-Fluo-4 AM probe was purchased from biolite Biotechnology (Xian, China). The 4-Phenylbutyric acid (4-PBA) was purchased from MedChemExpress (NJ, USA). Acridine orange (AO) dye was purchased from Solarbio (Beijing, China).

### The physicochemical characterizations of N-GQDs

The N-GQDs (Product No. XF241) used in this study was purchased from XFNANO Materials Tech Co., Ltd. (Nanjing, China) (http://www.xfnano.com), and their physicochemical characterizations were evaluated before the study. High-resolution transmission electron microscope (HR-TEM, JEM-2100, JEOL Ltd. Japan) images were acquired on an electron microscope. The particle morphology was examined by atomic force microscopy (AFM, Dimension Icon, Bruker AXS, German). The FT-IR spectra were recorded on Nicolet iS10 spectrometer (Thermo, USA). The absorption spectra and fluorescence spectra were measured on a UV-2550 spectrometer (Shimadzu, Japan). The dynamic light scattering (DLS) and surface ξ-potential measurements were carried out on a Malvern Zetasizer Nano ZS instrument (Zetasizer Nano-ZS90, Malvern, UK).

### Endotoxin testing

An end-point Limulus Amebocyte Lysate (LAL) assay was used to detect the Gram negative bacterial endotoxin in 100 µg/mL N-GQDs preparation according to the revised protocol based on Nanotechnology Characterization Laboratory (NCL) Method STE-1.2 [53]. As a preliminary indication of the interference of QDs with the LAL assay components, the recovery rate was calculated with the following formula [54]. It is considered acceptable in a range between 50 and 200% according to regulatory guidelines. All treatments were performed in triplicate in three independent experiments.

$$\begin{aligned} {\text{Recovery}}\;{\text{rate}}\;(\% ) & = \frac{{{\text{EU}}_{{{\text{sample}} + {\text{spiked}}\;{\text{endotoxin}}}} { } - {\text{EU}}_{{{\text{sample}}}} }}{{{\text{EU}}_{{{\text{spiked}}\;{\text{endotoxin}}}} }} \\ & \quad \times 100\;\;({\text{endotoxin}}\;{\text{spike}} = 0.{1}\;{\text{EU}}/{\text{mL}}) \\ \end{aligned}$$

### Animals and treatment

Total 30 male ICR mice (6 mice per group) aged 8 weeks were purchased from Zhejiang Academy of Medical Sciences (Zhejiang, China). All mice were housed in stainless steel cages in a ventilated animal facility at a temperature maintained in 22 ± 2 °C and relative humidity of 65 ± 10% under a 12 h light/dark cycle, and feed with sterilized food and distilled water. All mice were treated humanely throughout the experimental period. All animal procedures were performed in strict accordance with the Guidelines for Care and Use of Laboratory Animals of Southeast University and experiments were approved by the Animal Experimental Ethics Committee of Southeast University (Nanjing, China).

In N-GQDs-treatment groups, each animal was administered with 0.1 or 1 mg/kg BW N-GQDs through intranasal instillation for 28 consecutive days, while the control were exposed to normal saline solution with the same administration method and time. In Fer-1-treatment groups, each animal was administered with 5 mg/kg BW Fer-1 through intraperitoneal injection every fourth day for 28 days, meanwhile all animals were exposed to 1 mg/kg BW N-GQDs or saline through intranasal instillation every second day.

All mice were weighted throughout the whole exposure period and then sacrificed through inhaling carbon dioxide at the end of exposure. Blood of each animal was collected to test various blood cells using a routine blood test instrument (RJ-0C107223, Mindray, Shenzhen, China). The brains were quickly removed and weighted. A part of brain tissues were fixed in 4% (w/v) paraformaldehyde for 24 h, embedded in paraffin and then cut into slices of 5 µm thick to do histological, immunohistochemistry (IHC) and Immunofluorescence (IF) analyses. Hippocampi were dissected on ice and them fixed in 2.5% glutaraldehyde for TEM examination. Meanwhile, some dissected hippocampi were stored in liquid nitrogen and then was ground. The supernatant was taken to do lipid peroxidation MDA assay, NADP^+^/NADPH assay and iron assay according to the manufacturer’s instructions, and qRT-PCR analysis and western blotting analysis that were described as below.

### Pathological examinations

The brain sections were established using standard laboratory procedures, and then stained with hematoxylin and eosin (H&E), toluidine blue, and prussian blue enhanced with 3,3′-diaminobenzidine (DAB). All sections were evaluated using a light microscopy and images were taken. Ultrathin sections of hippocampus were established using standard laboratory procedures and observed by a TEM.

### Immunohistochemistry

The brain sections were incubated with 4-HNE and GRP78 primary antibodies at 1:100 dilution overnight at 4 °C. After that, slices were incubated with biotinylated anti-rabbit IgG secondary antibody at 1:500 dilution followed with DAB staining for signal development. All sections were visualized under a light microscope and images were taken.

### Immunofluorescence

The frozen brain sections were incubated with IBA1primary antibodies at 1:300 dilution overnight at 4 °C. After that, slices were incubated with Cy3 conjugated anti-mouse IgG at 1:500 dilution for 1 h at room temperature, and then stained by DAPI dye. The dead cells in hippocampus were detected by terminal-deoxynucleotidyl transferase mediated nick end labelling (TUNEL) analysis. All sections were visualized with a confocal microscope. The number of Iba1 and TUNEL positive cells were counted in 5 non-overlapping randomly selected fields in the CA1, CA3 and DG regions of each section.

### Cell lines and treatment

Microglial BV2 cell line was obtained from the Shanghai Cell Research Center (Shanghai, China) and maintained in our laboratory using Dulbecco’s modified Eagle’s medium (DMEM) high glucose medium supplemented with 10% fetal bovine serum (FBS) and 100 U/mL Penicillin. The cells were maintained in a humidified atmosphere of 95% O_2_ and 5% CO_2_ at 37 °C and the medium was changed in every 2 days. The exposure doses of N-GQDs were chosen as 25, 50 and 100 µg/mL, which were based on the previous study and applied concentration in the field of biomedicine [11, 12, 17]. For pre-treatment of inhibitor groups, a specific ferroptosis inhibitor Fer-1, an iron chelator DFOM, an intracellular calcium chelator BAPTA-AM, an inhibitor of calcium channel proteins in sarcoplasm/ER Dantrolene, three types of inhibitors of L-VGCCs nifedipine, diltiazem and verapamil, and a ER stress inhibitor 4-PBA were added to cells for 2 h prior to 100 µg/mL N-GQDs addition and maintained in the media until the exposure time was over at a final concentration of 500 nmol, 5 µmol, 20 µmol, 10 µmol, 10 µmol, 2.5 µmol, 10 µmol and 1 mmol.

### Cell viability and cell death assays

The cell viability of BV2 cells was determined using the cell counting kit-8 (CCK8) assay. Briefly, cells were cultured in 96-well plates and treated with N-GQDs at different concentrations for 24 h after completely attached to the bottom area. 10 µL water-soluble WST-8 [2-(2-methoxy-4-ni-trophenyl)-3-(4-nitrophenyl)-5-(2,4-disulfophenyl)-2H-tetrazolium, monosodium salt] solution was added into each well for 1 h at 37 °C and then measured spectrophotometric data using a multi-volume spectrophotometer system (Epoch, BioTek, USA) at a wavelength of 450 nm. The necrotic cells in BV2 cells were detected by PI dye using a flow cytometry (FACSCanto II; BD Bioscience). Briefly, exposed cells were collected and treated with a PI solution diluted in fresh normal cell culture medium at a final concentration of 1 µM and incubated at 37 °C for 30 min in dark. Finally, the labelled cells were washed with PBS before recording. All the treatments were performed in triplicate in three independent experiments based on the manufacturer’s instructions.

### Measurements of cytosolic and lipid ROS

Similar to the procedure of cell death assay, BV2 cells were collected and loaded with 1 µM DCFH-DA probe or 1 µM C11-BODIPY^581/591^ probe at 37 °C for 30 min in dark, and then labelled cells were washed with PBS and recorded by the flow cytometry.

### Microarray assay

BV2 cells treated with 100 µg/mL N-GQDs were designed for detecting the differentially expressed genes. Total RNAs extracted by Trizol reagent was quantified and assessed by a NanoDrop ND-2000 spectrophotometer (Thermo Scientific) at A260/A280 nm, and an Agilent Bioanalyzer 2100 (Agilent Technologies), respectively. The microarray analysis was completed by OE Biotech. Co., Ltd (Shanghai, China), and the sample labelling, microarray hybridization and washing were strictly performed in accordance with standard protocols. The detailed process is described in a previous study [28].

### ELISA assay

The intracellular levels of reduced glutathione (GSH) and oxidized glutathione (GSSG) in BV2 cells were determined by using commercial GSH/GSSG assay kit. Briefly, protein removal reagent M solution in the kit was added into the supernatants of tissue and cell homogenate and fully vortexed. After centrifugating for 10 min, the supernatant was used for the determination of total glutathione. Part of the prepared sample was added and vortexed with GSH removal auxiliary solution, and then GSH removal working solution in the kit for the determination of GSSG. Prepared two kinds of samples were measured using a multi-volume spectrophotometer system (Epoch, BioTek, USA) at 405 nm, and the GSH = total glurathione − GSSG × 2.

The MDA content in hippocampus and cells was measured using the commercial lipid peroxidation MDA assay kit. Briefly, thiobarbituric acid (TBA) in the kit was added to the supernatants of tissue and cell homogenate and then oscillated for the formation of TBA-MDA mixture that was determined at 535 nm using a multi-volume spectrophotometer system (Epoch, BioTek, USA).

The contents of NADP+ and NADPH in hippocampus were determined by the NADP+/NADPH assay kit. Briefly, NADP+/NADPH extracting solution in the kit was added into cells or tissues and homogenized. After centrifuging for 10 min, the supernatant was used for the determination of total content of NADP+ and NADPH (NADP_total_). Part of the prepared sample was put in a water bath at 60 °C for 30 min to decompose NADP+, and then incubated with G6PDH working solution in the kit at 37 °C for 30 min. After that, 10 µL chromogenic solution was added into each well for 10 min to determine NADPH content. Prepared two kinds of samples were measured using a multi-volume spectrophotometer system (Epoch, BioTek, USA) at 450 nm, and the NADP+ = NADP_total_-NADPH. All treatments were performed in triplicate in three independent experiments according to the manufacturer’s instructions.

### Iron detection

The frozen brain sections of 8 mm thickness were incubated with 5 µM FerroOrange for 30 min at 37 °C in a dark chamber to detect ferrous iron in hippocampus. After incubation, FerroOrange was removed by washing thrice with PBS and fluorescence images were obtained using a confocal microscope (FV1000, Olympus, Japan). Meanwhile, the levels of total iron, ferrous iron and ferric iron in tissue grinding fluid of hippocampus were determined by the commercial Iron Assay Kit with Colorimetric according to the manufacturer’s protocol using a multi-volume spectrophotometer system (Epoch, BioTek, USA) at the wavelength of 593 nm. The BV2 cells on glass bottomed dishes were incubated with 1 µM FerroOrange for 30 min at 37 °C to detect ferrous iron using the confocal microscope. All the treatments were performed in triplicate in three independent experiments.

### Calcium detection

Calcium iron contents in supernatants of tissue homogenate of hippocampus were measured by a calcium colorimetric assay kit according to the manufacturer’s protocol using a multi-volume spectrophotometer system (Epoch, BioTek, USA) at the wavelength of 575 nm. To detect the calcium in plasma and ER, the BV2 cells on glass bottomed dishes were incubated with 1 µM Fluo-4 AM and 5 µM Mag-Fluo-4 AM for 30 min at 37 °C in the dark to detect ferrous iron using the confocal microscope, respectively. All the treatments were performed in triplicate in three independent experiments.

### Western blotting analysis

The expression levels of protein in hippocampus and BV2 cells were assessed by western blotting analysis after extracting total protein. The proteins were electrophoresized in a 10% sodium dodecyl sulfatepolyacrylamide gelelectrophoresis (SDS-PAGE) separation gel, transferred to PVDF membranes and then blocked in 5% non-fat milk at room temperature for 1 h. After that, membranes were washed with Tris buffered saline tween (TBST) and then incubated with primary antibodies at 4 °C overnight. After washed with TBST, membranes were subsequently incubated with secondary antibodies at room temperature for 2 h. Finally, enhanced chemiluminenscence (ECL) solution (Millipore, USA) was used to exhibit protein bends. Protein levels were normalized to reference protein GAPDH. The same samples were ran three times and the experiment was repeated independently at least three times.

### Quantitative real-time PCR (qRT-PCR) analysis

Equal quantities of total RNA in hippocampus and BV2 cells were extracted using TRIzol (Invitrogen, USA), and then cDNA was synthesized by a reverse transcriptase rection using a Mastercycler gradient PCR system (Eppendorf, USA) and 1 µg/sample cDNA were used to do the qRT-PCR analysis. Briefly, a StepOnePlusTM real-time PCR system (Version 2.2.2, Applied Biosystems, USA) was used with the SYBR Green qRT-PCR master mix (TOYOBO, Japan). The qRT-PCR primers were designed by software Primer Premier based on National Center for Biotechnology Information (NCBI) (Additional file 1: Table S3). The relative quantities of mRNA were normalized to reference gene gapdh. Three replicates were conducted for each qRT-PCR analysis.

### Data analysis

All data were displayed as the mean ± standard deviation (SD). Statistical analysis was performed using Graphpad Prism 6.0 Software. One-way analysis of variance (ANOVA) was used to determine the statistical significance between the control and exposed groups followed with the Tukey LSD (Least Significant Difference) *post-hoc* test to determine the significance of differences between groups. Probability levels of < 0.05, < 0.01 and < 0.001were considered statistically significant.

## Supplementary Information

**Additional file 1: Supporting information of nitrogen-doped graphene quantum dots induce ferroptosis through disrupting calcium homeostasis in microglia. Table S1:** The summary of physicochemical characteristics of N-GQDs. **Table S2:** Effects of N-GQDs on routine blood indicators of mice. **Table S3:** Designed qRT-PCR primers of genes. **Figure S1:** The content of endotoxin in 100 µg/mL N-GQDs. **Figure S2:** The transportation and distribution of N-GQDs in hippocampus. **Figure S3:** The general toxic effects caused by N-GQDs in mice. **Figure S4:** N-GQDs induced impairments of hippocampus that was alleviated by Fer-1. **Figure S5:** Fer-1 reversed lipid peroxidation caused by N-GQDs in hippocampus. **Figure S6:** Pre-treatment of Fer-1 reversed lipid peroxidation caused by N-GQDs in BV2 cells. **Figure S7:** Representative fluorescent images of intracellular calcium level in BV2 cells. **Figure S8:** Pre-treatment of L-VGCCs inhibitors reversed cell damages and inflammation caused by N-GQDs in BV2 cells. **Figure S9:** Pre-treatment of L-VGCCs inhibitors alleviated lipid peroxidation caused by N-GQDs in BV2 cells. **Figure S10:** Pre-treatment of RyR channels inhibitor reversed cell damages and inflammation caused by N-GQDs in BV2 cells. **Figure S11:** Pre-treatment of RyR channels inhibitor alleviated lipid peroxidation caused by N-GQDs in BV2 cells. **Figure S12:** N-GQDs caused ER stress response in hippocampus and BV2 cells. **Figure S13:** N-GQDs caused the alternation of genes associated with ER stress in BV2 cells. **Figure S14:** The distribution of N-GQDs in cells. **Figure S15:** The protein expressions of BIP, CHOP and ATF4.

## Data Availability

The datasets during and/or analyzed during the current study available from the corresponding author on reasonable request.
