# Supplementary material for: Nitrogen-doped graphene quantum dots induce ferroptosis through disrupting calcium homeostasis in microglia
Source: Part Fibre Toxicol. 2022 Mar 24;19:22. doi: 10.1186/s12989-022-00464-z (PMC8944010; doi:10.1186/s12989-022-00464-z)
Supplement: Supplementary file 1 — Additional file 1: Supporting information of nitrogen-doped graphene quantum dots induce ferroptosis through disrupting calcium homeostasis in microglia. Table S1: The summary of physicochemical characteristics of N-GQDs. Table S2: Effects of N-GQDs on routine blood indicators of mice. Table S3: Designed qRT-PCR primers of genes. Figure S1: The content of endotoxin in 100 µg/mL N-GQDs. Figure S2: The transportation and distribution of N-GQDs in hippocampus. Figure S3: The general toxic effects caused by N-GQDs in mice. Figure S4: N-GQDs induced impairments of hippocampus that was alleviated by Fer-1. Figure S5: Fer-1 reversed lipid peroxidation caused by N-GQDs in hippocampus. Figure S6: Pre-treatment of Fer-1 reversed lipid peroxidation caused by N-GQDs in BV2 cells. Figure S7: Representative fluorescent images of intracellular calcium level in BV2 cells. Figure S8: Pre-treatment of L-VGCCs inhibitors reversed cell damages and inflammation caused by N-GQDs in BV2 cells. Figure S9: Pre-treatment of L-VGCCs inhibitors alleviated lipid peroxidation caused by N-GQDs in BV2 cells. Figure S10: Pre-treatment of RyR channels inhibitor reversed cell damages and inflammation caused by N-GQDs in BV2 cells. Figure S11: Pre-treatment of RyR channels inhibitor alleviated lipid peroxidation caused by N-GQDs in BV2 cells. Figure S12: N-GQDs caused ER stress response in hippocampus and BV2 cells. Figure S13: N-GQDs caused the alternation of genes associated with ER stress in BV2 cells. Figure S14: The distribution of N-GQDs in cells. Figure S15: The protein expressions of BIP, CHOP and ATF4. [file 12989_2022_464_MOESM1_ESM.docx]

**Supporting information**

Nitrogen-doped graphene quantum dots induce ferroptosis through disrupting calcium homeostasis in microglia

Tianshu Wu^1,*^, Xinyu Wang^1^, Jin Cheng^1^, Xue Liang^2^, Yimeng Li^1^, Min Chen^1^, Lu Kong^1^, and Meng Tang^1^

^1^ Key Laboratory of Environmental Medicine and Engineering, Ministry of Education; School of Public Health, Southeast University, Nanjing 210009, P.R.China.

^2^ School of Public Health, Shandong First Medical University & Shandong Academy of Medical Sciences, Jinan, P.R.China.

^*^ Author to whom correspondence should be addressed. E-Mail: [ninatswu@126.com](mailto:ninatswu@126.com), [ninatswu@seu.edu.cn](mailto:ninatswu@seu.edu.cn)

**Table S1:** The summary of physicochemical characteristics of N-GQDs

| QDs | Mean size by TEM (nm) | Thickness (nm) | Mean size by DLS in DI water (nm) | Mean size by DLS in DMEM (nm) | ξ-potential in DI water (mV) | ξ-potential in DMEM (mV) | Excitation peak in DI water (nm) | Excitation peak in DMEM (nm) | Emission peak in DI water (nm) | Emission peak in DMEM (nm) | PLQY (%) |
| --- | --- | --- | --- | --- | --- | --- | --- | --- | --- | --- | --- |
| N-GQDs | ~3 | 0.5~3 | 4.6 | 5.2 | -9.9 | -12.2 | 350 | 380 | 420 | 480 | ~20 |

**Table S2:** Effects of N-GQDs on routine blood indicators of mice. Data are expressed as the mean ± SE of three independent experiments, performed in triplicate. Statistical significance was determined by one-way ANOVA and Tukey LSD post-hoc test (*P < 0.05, **P < 0.01 vs. the control group)

| **Group** | **Indicators** | | | | |
| --- | --- | --- | --- | --- | --- |
|  | **WBC (10^9^/L)** | **RBC (10^12^/L)** | **NEU (10^9^/L)** | **LY (10^9^/L)** | **MONO (10^9^/L)** |
| Ctrl | 3.72+0.42 | 1.23+0.23 | 2.28+0.34 | 0.15+0.03 | 0.05+0.01 |
| N-GQDs-0.1 | 3.84+0.52 | 1.08+0.12 | 2.64+0.23 | 0.11+0.02 | 0.07+0.01 |
| N-GQDs-1 | 6.39+1.34** | 2.02+0.31 | 3.88+0.36** | 0.4+0.01* | 0.09+0.02 |
| N-GQDs+Fer-1 | 3.55+0.92 | 1.59+0.14 | 1.49+0.24 | 0.09+0.01 | 0.08+0.01 |
| Ctrl+Fer-1 | 5.51+1.13* | 1.32+0.23 | 3.76+0.42** | 0.16+0.02 | 0.05+0.01 |

WBC, white blood count; RBC, red blood count; NEU, neutrophil cell; LY, lymphocyte; MONO, Monocyte

**Table S3:** Designed qRT-PCR primers of genes.

| **Gene name** | **Designed qRT-PCR primers** | |
| --- | --- | --- |
|  | Forward | Backward |
| ddit3 | CCTGAGGAGAGAGTGTTCCAG | GACACCGTCTCCAAGGTGAA |
| hspa5 | CGTGTGTGTGAGACCAGAAC | ACAGTGAACTTCATCATGCCG |
| igbp1 | CGCGTTCGGGAATATCACCT | AGTGACAAGCTGTTTCCTCTCT |
| atf4 | TAAGTTGTGTGCTCGGGTGT | GGATTTCGTGAAGAGCGCCAT |
| gapdh | CCTGTTCCAGAGACAGCCGC | GCGCCCAATACGGCCAAATC |

**Figure S1: The content of endotoxin in 100 µg/mL N-GQDs.**

**
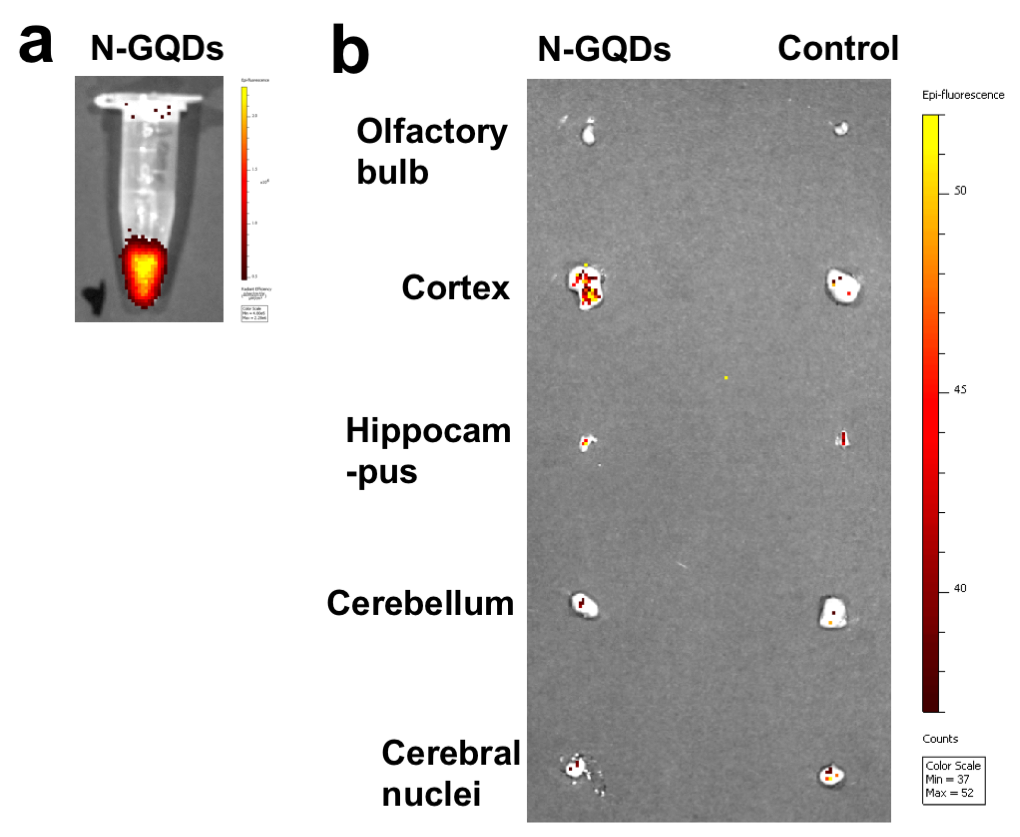
**

**Figure S2: The transportation and distribution of N-GQDs in hippocampus.** (a) Representative fluorescence pictures of N-GQDs; (b) Representative fluorescence pictures of the main areas of brain tissue in mice. Each mouse was intranasally instilled with saline and 1 mg/kg BW N-GQDs every other day for 28 days.

**
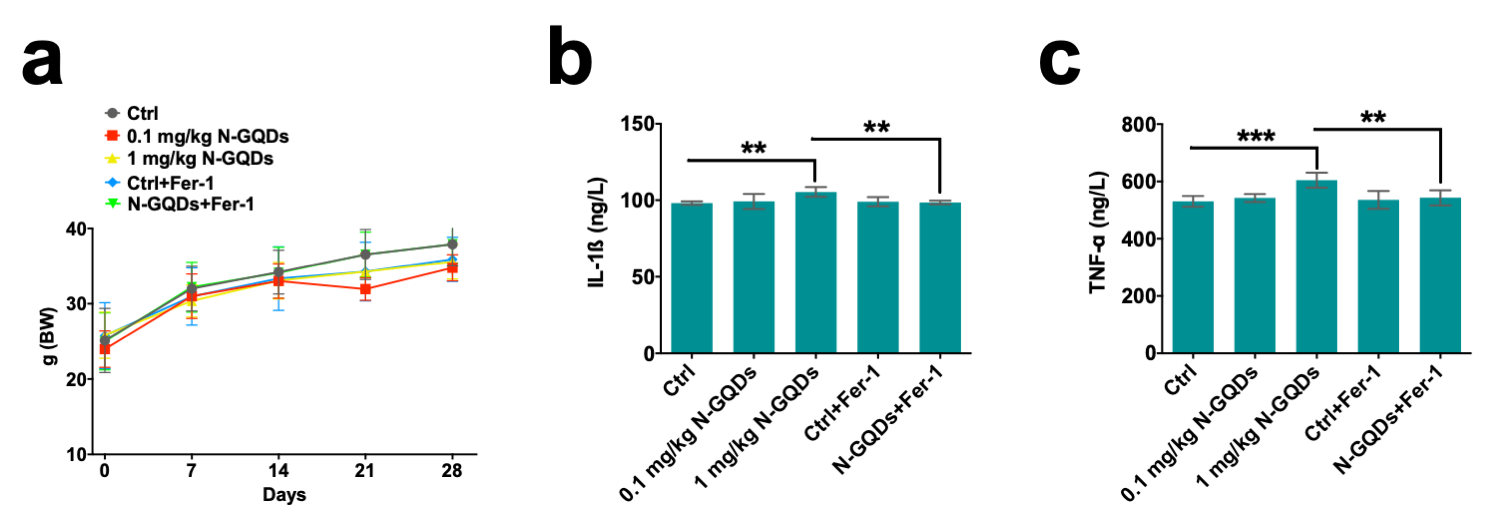
**

**Figure S3: The general toxic effects caused by N-GQDs in mice.** (a) The changes in body weight of mice; (b, c) The levels of IL-1ß and TNF-α in serum. Each mouse was intranasally instilled with saline, 0.1 and 1 mg/kg BW N-GQDs every other day for 28 days. For Fer-1 pre-treatment groups, each mouse was intraperitoneally injected with 5 mg/kg BW Fer-1 every fourth day and intranasally instilled with 1 mg/kg BW N-GQDs every other day for 28 days (mouse n=6). Data are showed as mean+SD of three independent experiments. The one-way ANOVA followed by the Dunnett’s t test were used to determine statistical significance (**P*<0.05, ***P*<0.01, ****P*<0.001 vs the control)


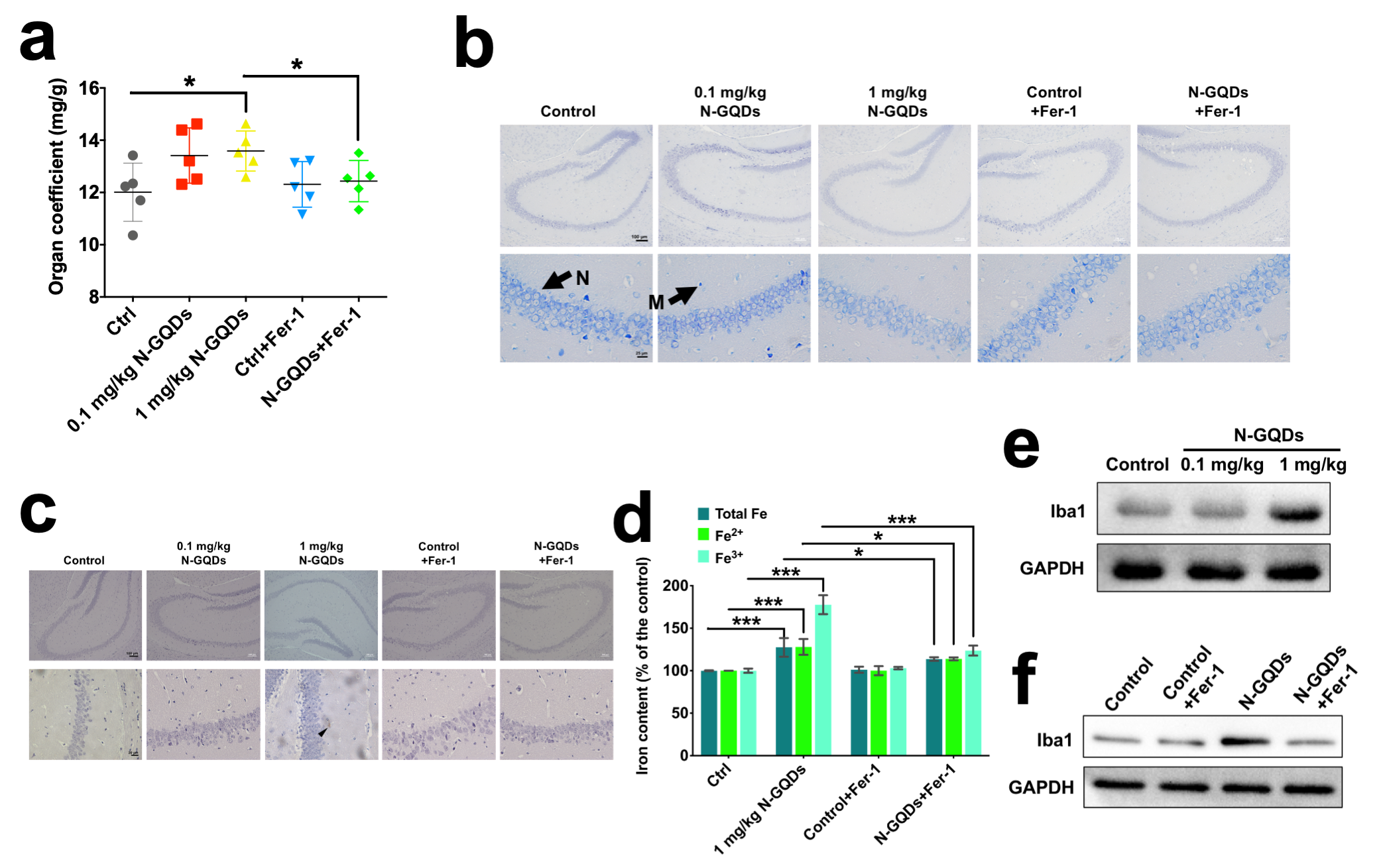


**Figure S4: N-GQDs induced impairments of hippocampus that was alleviated by Fer-1.** (a) The changes in organ coefficient of mice brain; (b) Representative histological images presented the hippocampus stained with toluidine blue. The neurons (N) contain Nissl bodies with a single nucleus in a large cell body, and microglia (M) was stained with irregular nuclei; (c) Representative histological images presented the hippocampus stained using Prussian blue intensificated with DAB. The arrows indicated deposited ferruginous substances displayed as tan color;

(d) The levels of total iron, ferrous iron (Fe^2+^) and ferric iron (Fe^3+^) in hippocampus; (e, f) The protein expressions of Iba1 in hippocampus using western blotting analysis. For merely N-GQDs exposure groups, each mouse was intranasally instilled with saline, 0.1 and 1 mg/kg BW N-GQDs every other day for 28 days. For Fer-1 pre-treatment groups, each mouse was intraperitoneally injected with 5 mg/kg BW Fer-1 every fourth day and intranasally instilled with 1 mg/kg BW N-GQDs every other day for 28 days (mouse n=6). Data are showed as mean+SD of three independent experiments. The one-way ANOVA followed by the Dunnett’s t test were used to determine statistical significance (**P*<0.05, ***P*<0.01, ****P*<0.001 vs the control and 1 mg/kg BW N-GQDs)


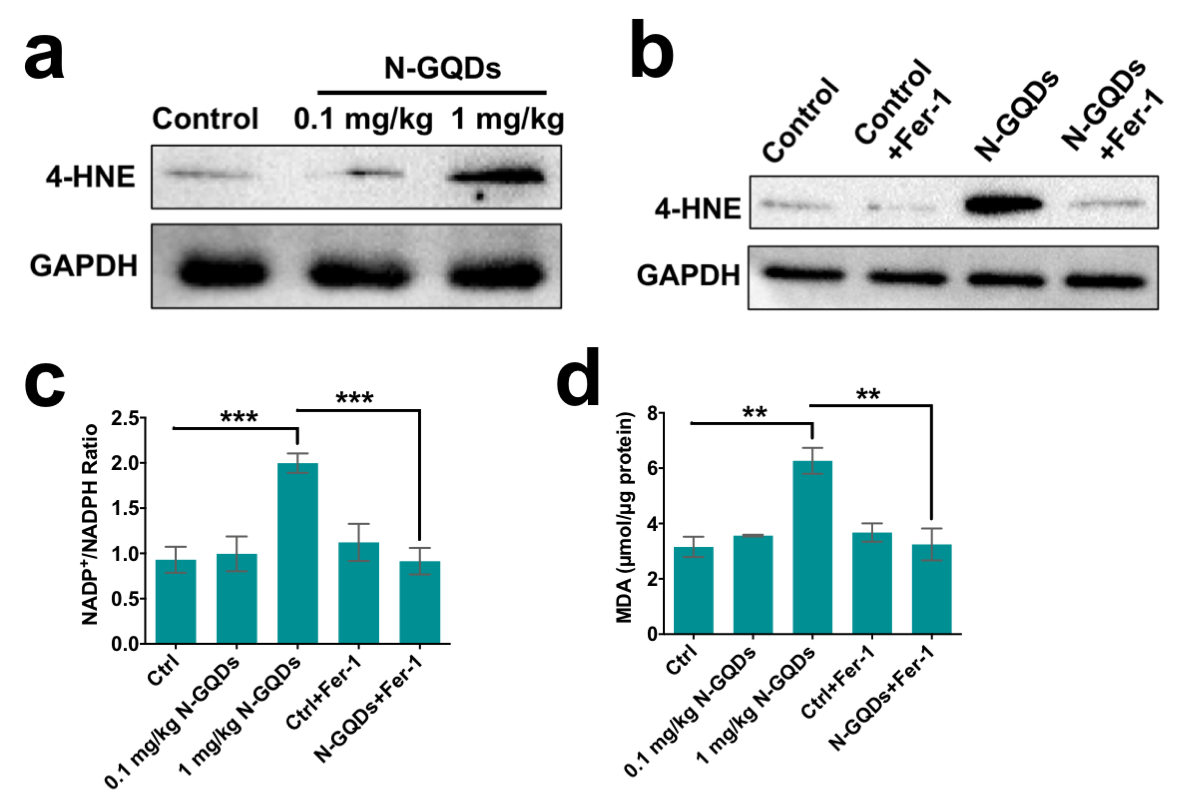


**Figure S5: Fer-1 reversed lipid peroxidation caused by N-GQDs in hippocampus.** (a, b) The protein expressions of 4-HNE in hippocampus using western blotting analysis; (c, d) NADP+/NADPH ratio and MDA content in hippocampus. For merely N-GQDs exposure groups, each mouse was intranasally instilled with saline, 0.1 and 1 mg/kg BW N-GQDs every other day for 28 days. For Fer-1 pre-treatment groups, each mouse was intraperitoneally injected with 5 mg/kg BW Fer-1 every fourth day and intranasally instilled with 1 mg/kg BW N-GQDs every other day for 28 days (mouse n=6). Data are showed as mean+SD of three independent experiments. The one-way ANOVA followed by the Dunnett’s t test were used to determine statistical significance (**P*<0.05, ***P*<0.01, ****P*<0.001 vs the control and 1 mg/kg BW N-GQDs)


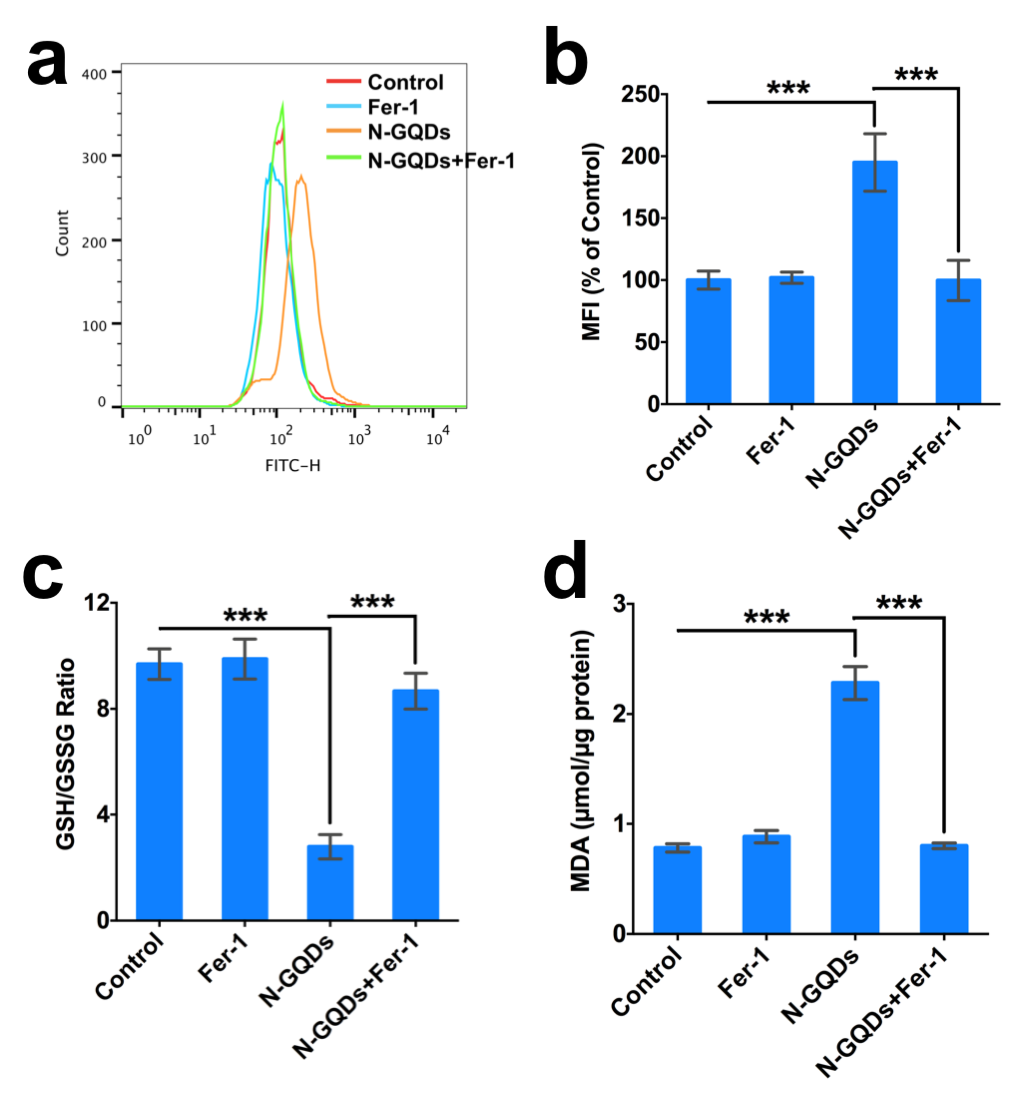


**Figure S6: Pre-treatment of Fer-1 reversed lipid peroxidation caused by N-GQDs in BV2 cells.** (a, b) Representative FITC fluorescence histogram plot of BV2 cells showed cytosolic ROS production using DCFH-DA dye, and the quantitative results of mean fluorescence intensity (MFI); (c, d) The GSH/GSSG ratio and MDA content in BV2 cells. BV2 cells were pre-treated with Fer-1 for 2 h and then exposed to 100 µg/mL N-GQDs for 24 h (n=3). Data are showed as mean+SD of three independent experiments. The one-way ANOVA followed by the Dunnett’s t test were used to determine statistical significance (**P*<0.05, ***P*<0.01, ****P*<0.001 vs the control or 100 µg/mL N-GQDs)


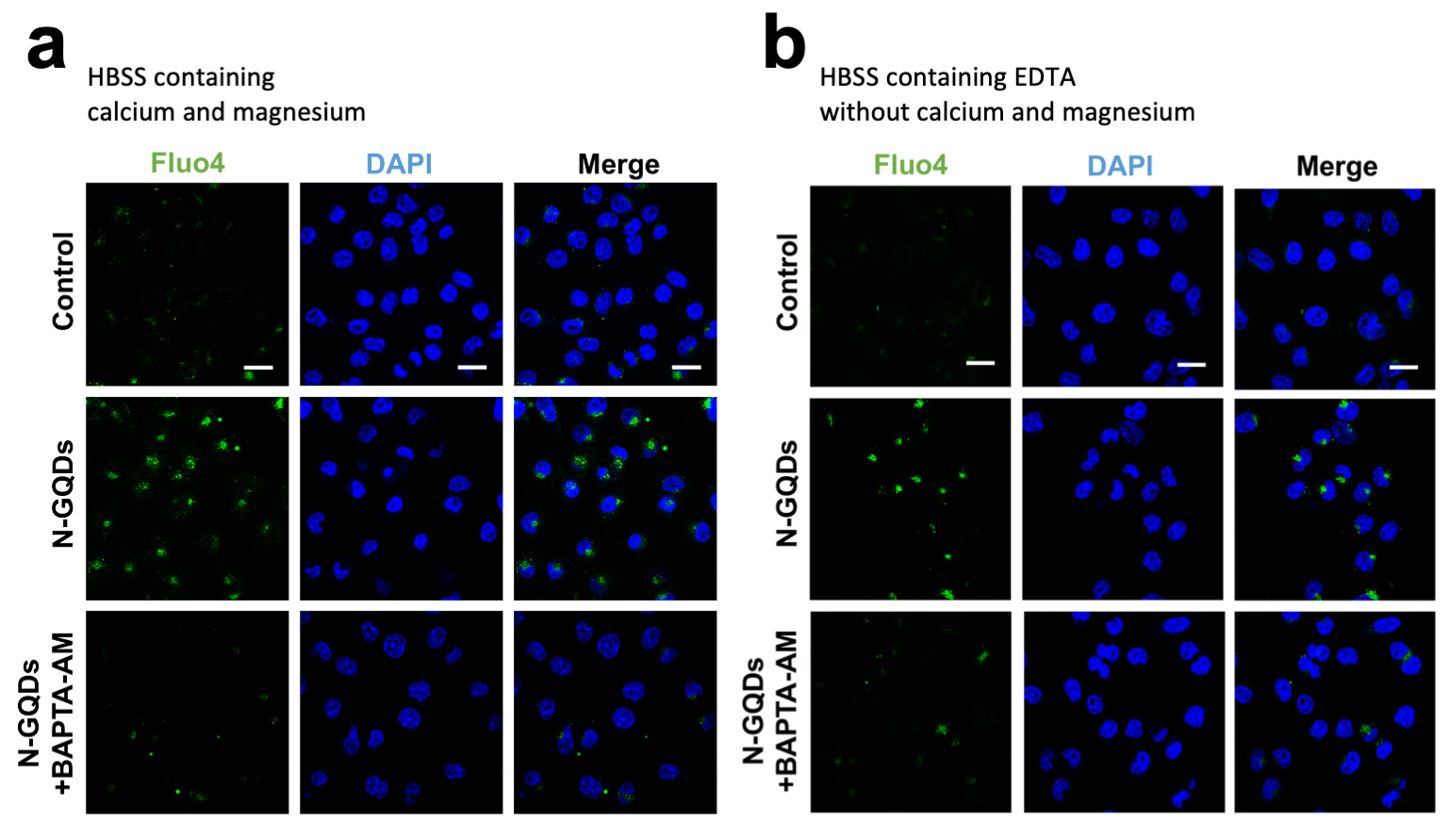


**Figure S7: Representative fluorescent images of intracellular calcium level in BV2 cells.** (a) Cells were maintained in HBSS containing calcium and magnesium (b) Cells were maintained in HBSS containing EDTA without calcium and magnesium. Scale bar: 20 µm. BV2 cells were treated with 100 µg/mL N-GQDs for 24 h when pre-treated with BAPTA-AM showing green using Fluo4 dye. The nucleus showed blue using DAPI. Scale bar: 20 µm


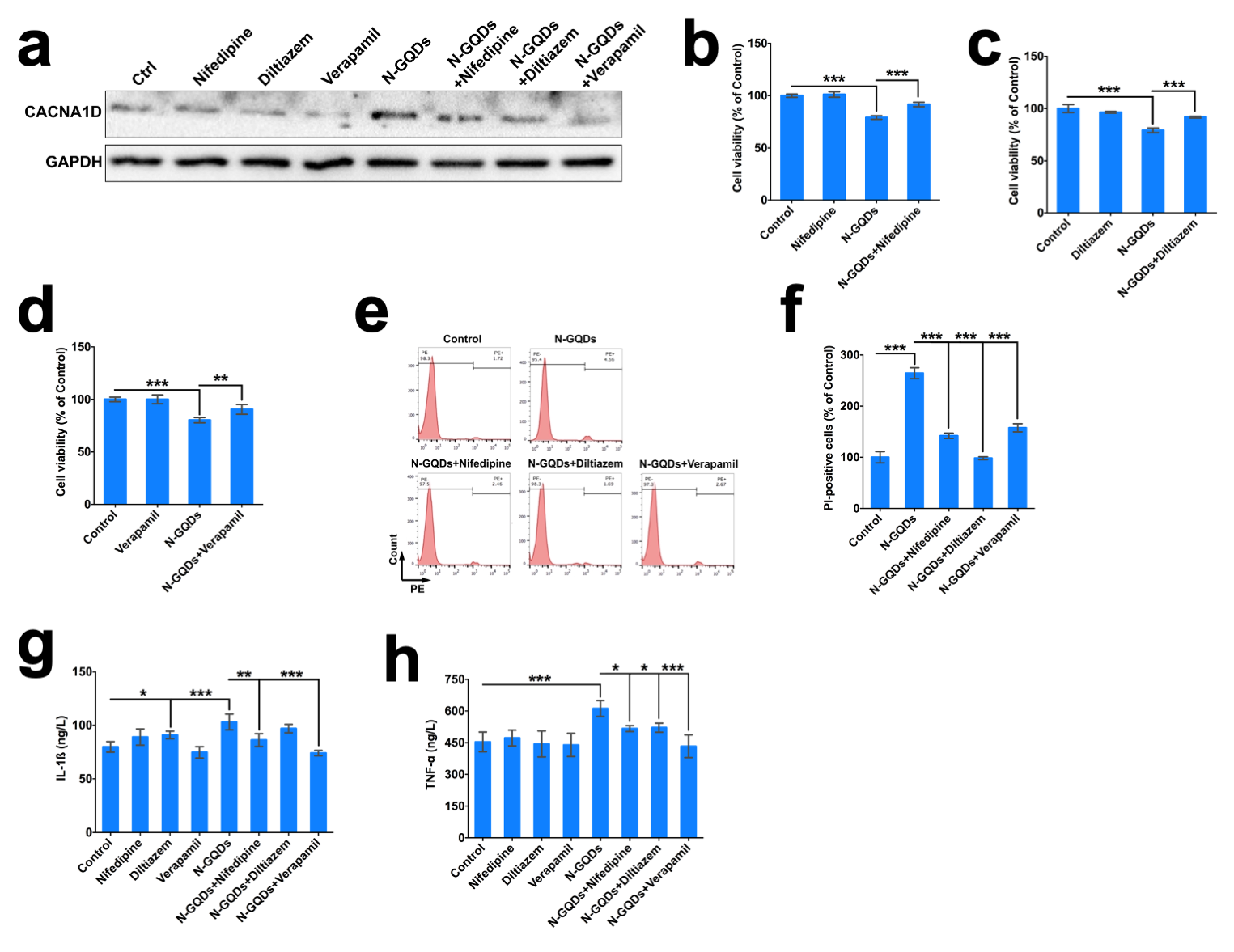


**Figure S8:** **Pre-treatment of L-VGCCs inhibitors reversed cell damages and inflammation caused by N-GQDs in BV2 cells.** (a) The protein expressions of CACNA1D in BV2 cells using western blotting analysis; (b, c, d) The cell viability of BV2 cells; (e, f) Representative PE fluorescence histogram plots of BV2 cells showed necrotic cells using PI dye, and the quantitative results of necrotic percentages; (g, h) The levels of IL-1ß and TNF-α in BV2 cells. BV2 cells were pre-treated with nifedipine, diltiazem and verapamil for 2 h and then exposed to 100 µg/mL N-GQDs for 24 h (n=3). Data are showed as mean+SD of three independent experiments. The one-way ANOVA followed by the Dunnett’s t test were used to determine statistical significance (**P*<0.05, ***P*<0.01, ****P*<0.001 vs the control or 100 µg/mL N-GQDs)

**
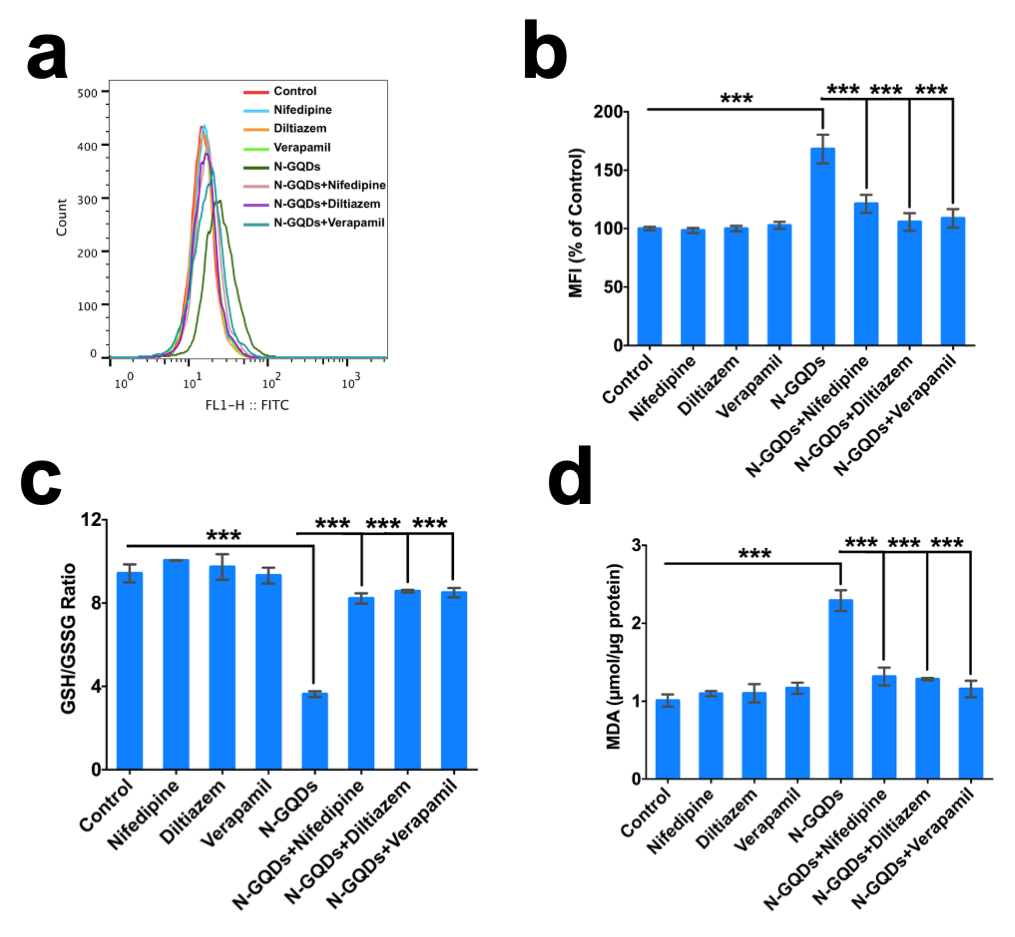
**

**Figure S9: Pre-treatment of L-VGCCs inhibitors alleviated lipid peroxidation caused by N-GQDs in BV2 cells.** (a, b) Representative FITC fluorescence histogram plot of BV2 cells showed lipid ROS using C11BODIPY581/591 dye, and the quantitative results of mean fluorescence intensity (MFI); (c, d) The GSH/GSSG ratio and MDA content in BV2 cells. BV2 cells were pre-treated with nifedipine, diltiazem and verapamil for 2 h and then exposed to 100 µg/mL N-GQDs for 24 h (n=3). Data are showed as mean+SD of three independent experiments. The one-way ANOVA followed by the Dunnett’s t test were used to determine statistical significance (**P*<0.05, ***P*<0.01, ****P*<0.001 vs the control or 100 µg/mL N-GQDs)


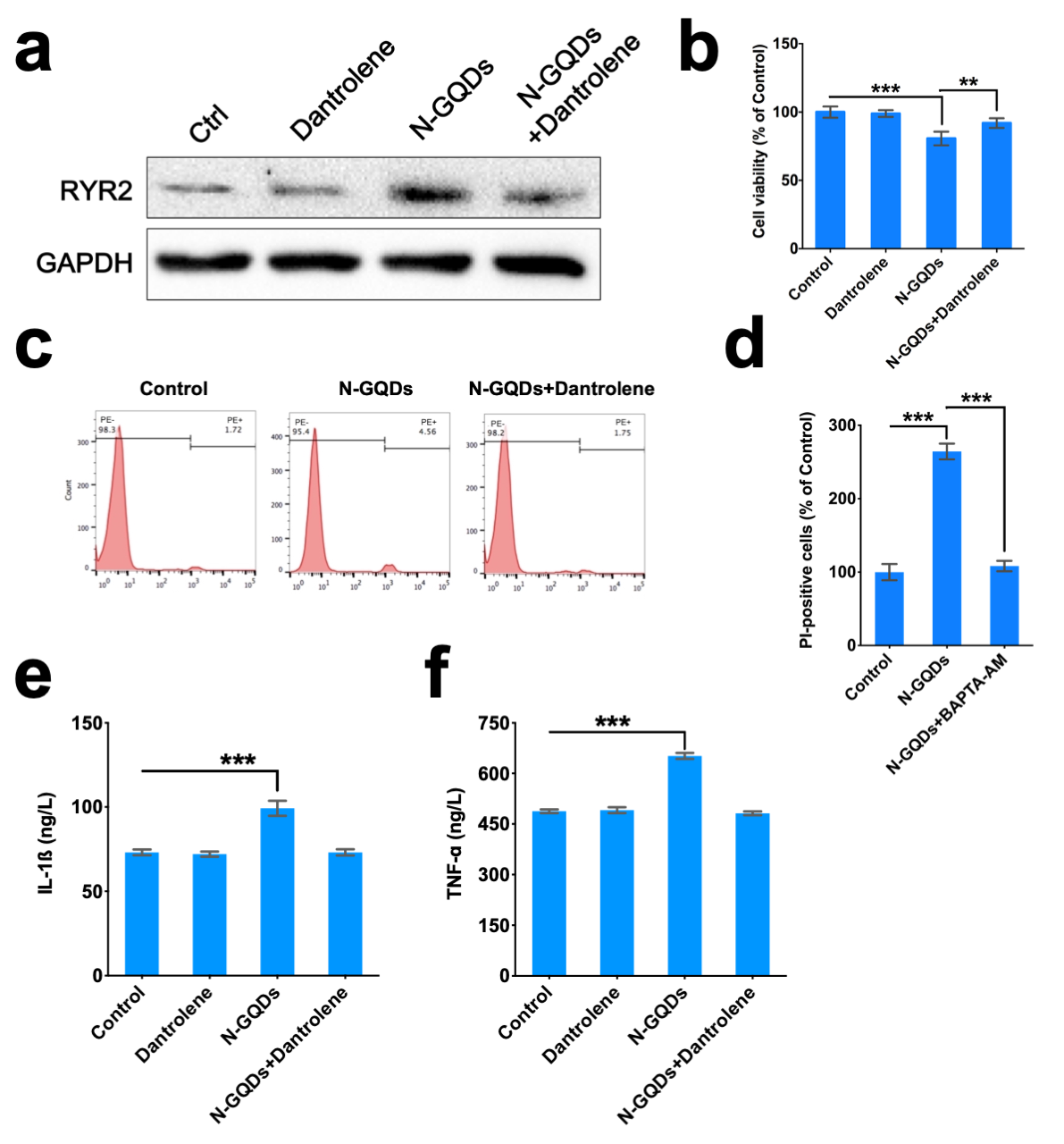


**Figure S10: Pre-treatment of RyR channels inhibitor reversed cell damages and inflammation caused by N-GQDs in BV2 cells.** (a) The protein expressions of RYR2 in BV2 cells using western blotting analysis; (b) The cell viability of BV2 cells; (c, d) Representative PE fluorescence histogram plots of BV2 cells showed necrotic cells using PI dye, and the quantitative results of necrotic percentages; (e, f) The levels of IL-1ß and TNF-α in BV2 cells. BV2 cells were pre-treated with dantrolene for 2 h and then exposed to 100 µg/mL N-GQDs for 24 h (n=3). Data are showed as mean+SD of three independent experiments. The one-way ANOVA followed by the Dunnett’s t test were used to determine statistical significance (**P*<0.05, ***P*<0.01, ****P*<0.001 vs the control or 100 µg/mL N-GQDs)


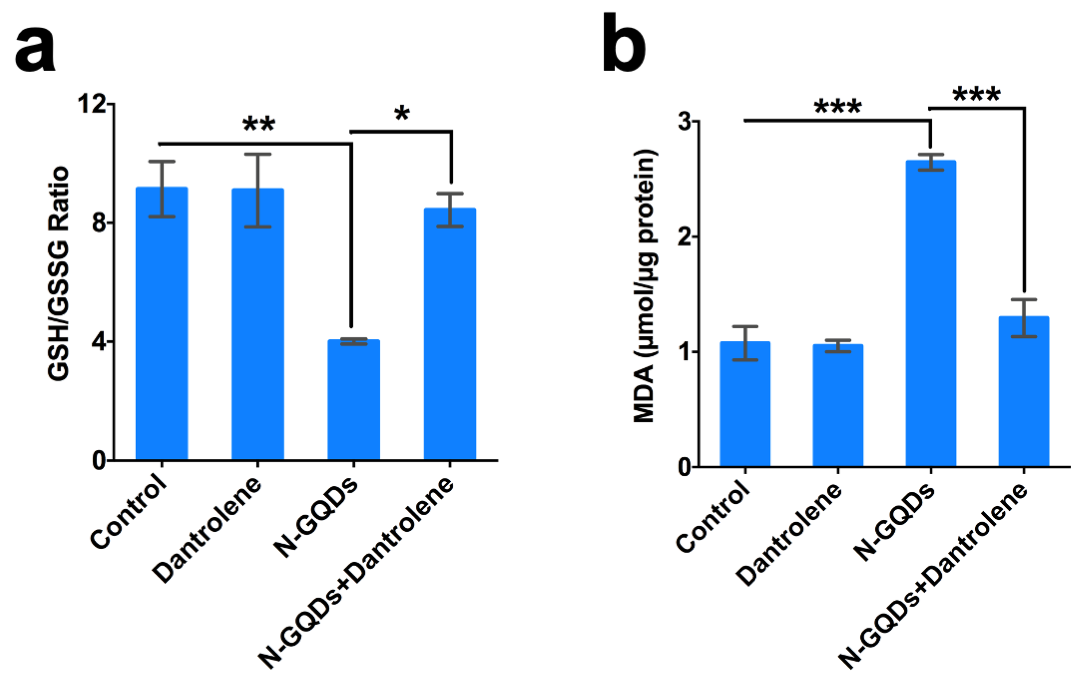


**Figure S11: Pre-treatment of RyR channels inhibitor alleviated lipid peroxidation caused by N-GQDs in BV2 cells.** (a, b) The GSH/GSSG ratio and MDA content in BV2 cells. BV2 cells were pre-treated with dantrolene for 2 h and then exposed to 100 µg/mL N-GQDs for 24 h (n=3). Data are showed as mean+SD of three independent experiments. The one-way ANOVA followed by the Dunnett’s t test were used to determine statistical significance (**P*<0.05, ***P*<0.01, ****P*<0.001 vs the control or 100 µg/mL N-GQDs)


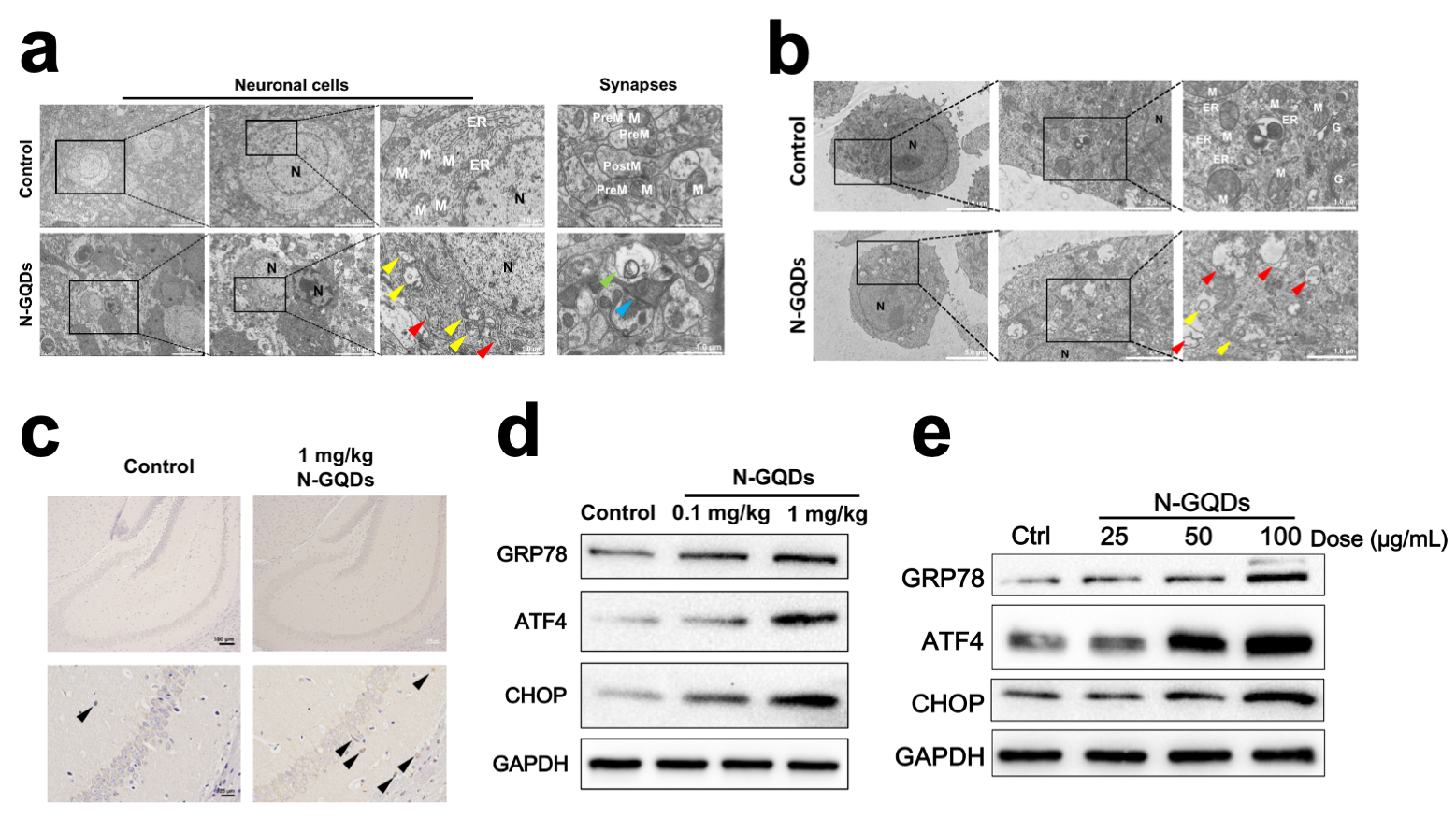


**Figure S12: N-GQDs caused ER stress response in hippocampus and BV2 cells.** (a) Representative TEM images showed the ultrastructure of neuronal cells in hippocampus. The red arrows indicate dilated endoplasmic reticulum, the yellow arrows indicate broken mitochondrial ridge, the blue arrow indicates a slight electron-dense degeneration in synapse, the green arrow indicates a swollen postsynaptic membrane. N (nucleus), M (mitochondria), ER (Endoplasmic reticulum), G (golgi), PreM (presynaptic membrane), postM (postsynaptic membrane); (b) Representative TEM images showing the ultrastructure of BV2 cells. The red arrows indicate dilated endoplasmic reticulum, the yellow arrows indicate broken mitochondrial ridge. N (nucleus), M (mitochondria), ER (Endoplasmic reticulum), G (golgi); (c) Representative immunohistochemical images presented GRP78 in hippocampus. The arrows indicate increased expression of GRP78 that displayed tan color; (d) The protein expressions of GRP78, CHOP and ATF4 in hippocampus using western blotting analysis; (e) The protein expressions of GRP78, CHOP and ATF4 in BV2 cells using western blotting analysis. Each mouse was intranasally instilled with saline, 0.1 and 1 mg/kg BW N-GQDs every other day for 28 days (mouse n=6). BV2 cells were treated with 25, 50 and 100 μg/mL N-GQDs for 24 h, or were pre-treated with dantrolene for 2 h and then exposed to 100 µg/mL N-GQDs for 24 h (n=3).


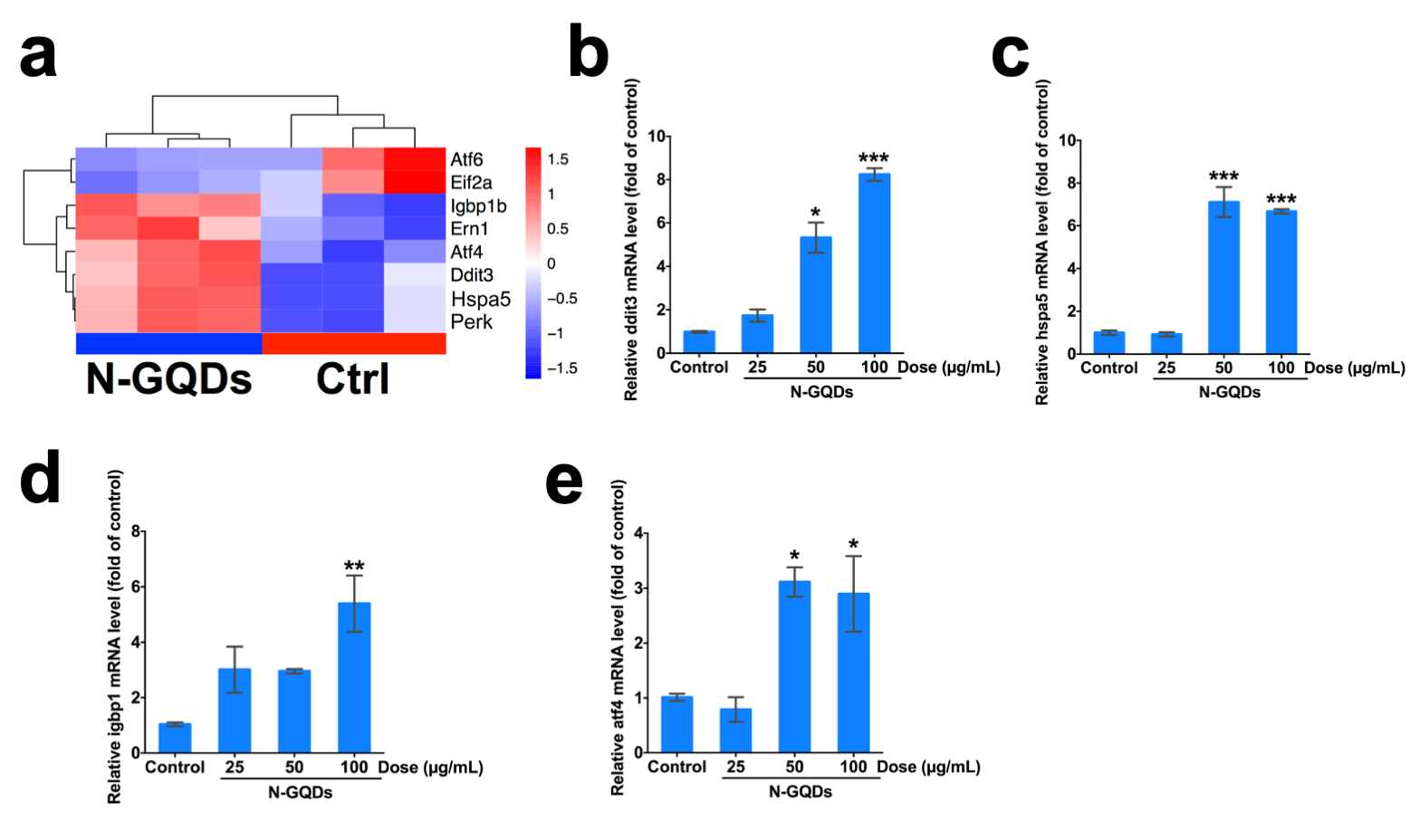


**Figure S13:** **N-GQDs caused the alternation of genes associated with ER stress in BV2 cells.** (a) Heatmap of differentially expressed genes associated with ER stress in BV2 cells treated with 100 µg/mL N-GQDs for 24 h using microarray analysis; (b, c, d, e) The gene expressions of ddit3, hspa5, igbp1 and atf4 in BV2 cells using qRT-PCR analysis. BV2 cells were treated with 25, 50 and 100 μg/mL N-GQDs for 24 h (n=3). Data are showed as mean+SD of three independent experiments. The one-way ANOVA followed by the Dunnett’s t test were used to determine statistical significance (**P*<0.05, ***P*<0.01, ****P*<0.001 vs the control)


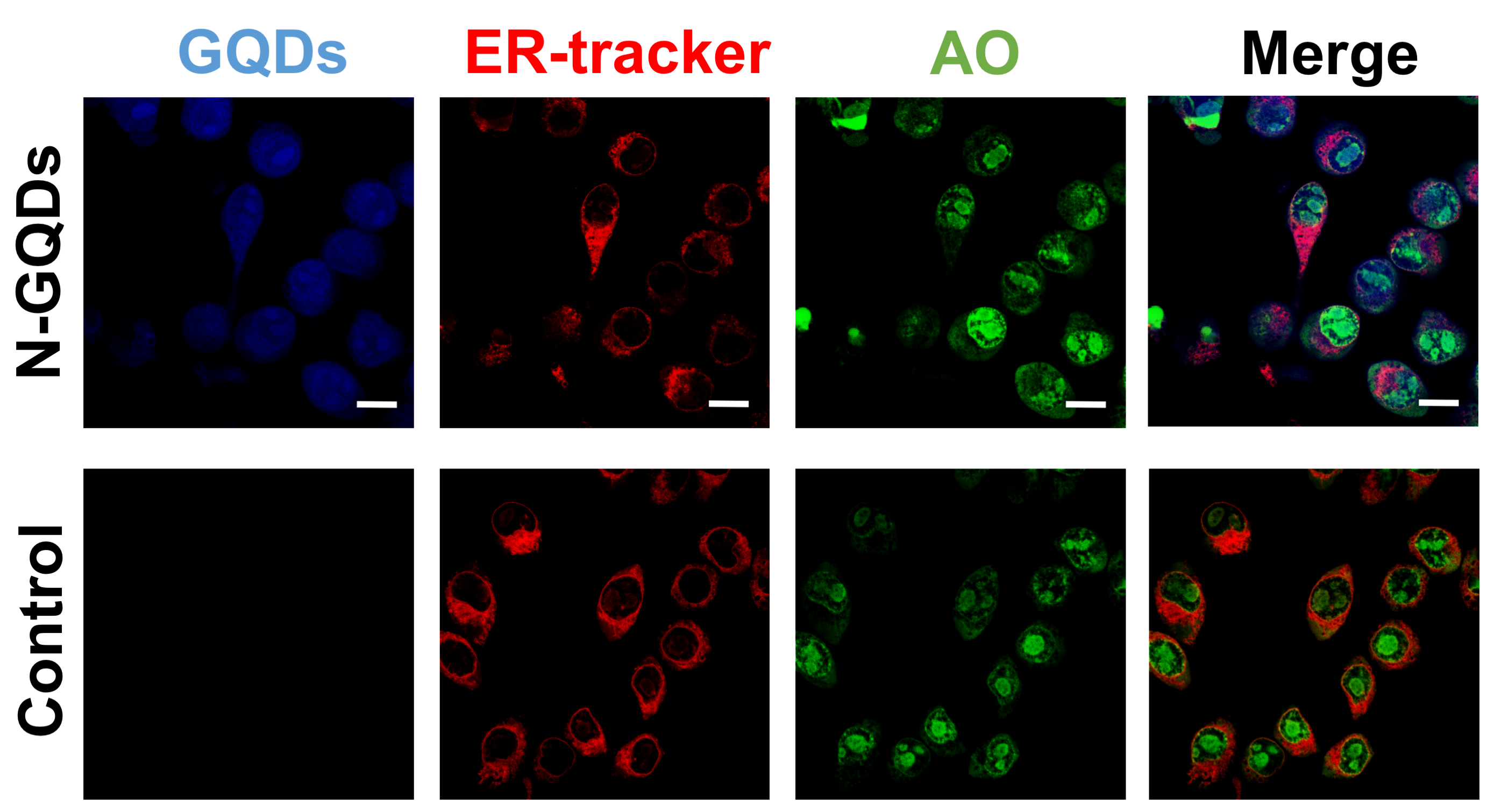


**Figure S14: The distribution of N-GQDs in cells.** Representative fluorescence images of N-GQDs in BV2 cells. The N-GQDs showed blue, the nucleus showed green using OA, and the ER showed red using ER-tracker Red. Scale bar: 20 µm. BV2 cells were exposed to 100 µg/mL N-GQDs for 24 h.


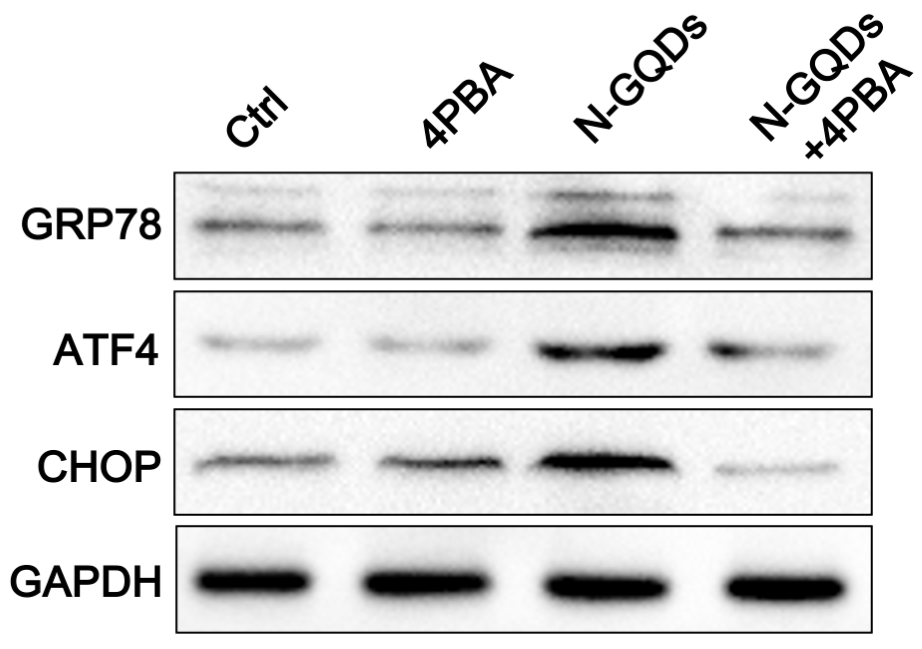


**Figure S15:** The protein expressions of BIP, CHOP and ATF4 in BV2 cells treated with 100 µg/mL N-GQDs for 24 h when cells were pre-treated with 4-PBA.
